# LncRNA MYLK-AS1 facilitates tumor progression and angiogenesis by targeting miR-424-5p/E2F7 axis and activating VEGFR-2 signaling pathway in hepatocellular carcinoma

**DOI:** 10.1186/s13046-020-01739-z

**Published:** 2020-11-09

**Authors:** Fei Teng, Ju-Xiang Zhang, Qi-Meng Chang, Xu-Bo Wu, Wei-Guo Tang, Jian-Fa Wang, Jin-Feng Feng, Zi-Ping Zhang, Zhi-Qiu Hu

**Affiliations:** 1 Department of Hepatobiliary and Pancreatic Surgery, Minhang Hospital, Fudan University, Shanghai, 201199 People’s Republic of China; 2 Institute of Fudan-Minhang Academic Health System, Minhang Hospital, Fudan University, Shanghai, 201199 People’s Republic of China; 3 Shanghai Med-X Engineering Center for Medical Equipment and Technology, School of Biomedical Engineering, Shanghai Jiao Tong University, Shanghai, 200030 People’s Republic of China

**Keywords:** Long non-coding RNAs, MYLK-AS1, Hepatocellular carcinoma, E2F7, Competing endogenous RNA, VEGFR-2, miR-424-5p

## Abstract

**Background:**

Long non-coding RNAs (lncRNAs) are crucial in the invasion, angiogenesis, progression, and metastasis of hepatocellular carcinoma (HCC). The lncRNA MYLK-AS1 promotes the growth and invasion of HCC through the EGFR/HER2-ERK1/2 signaling pathway. However, the clinical significance of MYLK-AS1 in HCC still needs to be further determined.

**Methods:**

Bioinformatic analysis was performed to determine the potential relationship among MYLK-AS1, miRNAs and mRNAs. A total of 156 samples of normal liver and paired HCC tissues from HCC patients were used to evaluate MYLK-AS1 expression by qRT-PCR. Human HCC cell lines were used to evaluate the colony formation, cell proliferation, migration, invasion, cell cycle and apoptosis after transfection of lentiviral short-hairpin RNAs (shRNAs) targeting MYLK-AS1 or MYLK-AS1 vectors. The competitive endogenous RNA (ceRNA) mechanism was clarified using fluorescence in situ hybridization (FISH), Western blotting, qPCR, RNA binding protein immunoprecipitation (RIP), and dual luciferase reporter analysis.

**Results:**

MYLK-AS1 up-regulation was detected in the HCC tumor tissues and cell lines associated with the enhancement of the angiogenesis and tumor progression. The down-regulation of MYLK-AS1 reversed the effects on angiogenesis, proliferation, invasion and metastasis in the HCC cells and in vivo. MYLK-AS1 acted as ceRNA, capable of regulating the angiogenesis in HCC, while the microRNA miR-424-5p was the direct target of MYLK-AS1. Promoting the angiogenesis and the tumor proliferation, the complex MYLK-AS1/miR-424-5p activated the VEGFR-2 signaling through E2F7, whereas the specific targeting of E2F transcription factor 7 (E2F7) by miR-424-5p, was indicated by the mechanism studies.

**Conclusions:**

MYLK-AS1 and E2F7 are closely related to some malignant clinicopathological features and prognosis of HCC, thus the MYLK-AS1/ miR-424-5p/E2F7 signaling pathway might represent a promising treatment strategy to combat HCC.

**Supplementary information:**

**Supplementary information** accompanies this paper at 10.1186/s13046-020-01739-z.

## Background

Hepatocellular carcinoma (HCC) is the sixth most common malignant tumor worldwide, with a very poor prognosis [1]. The biggest obstacle affecting the diagnosis and treatment of HCC is its high metastatic rate and invasion ability inside and outside the liver, and strong angiogenetic ability [2]. Therefore, despite many articles are available regarding the regulation of HCC, a further and deeper study on the mechanisms regulating HCC metastasis, invasion and angiogenesis is urgently needed.

Long non-coding RNA (lncRNA) is a non-coding RNA with a length of more than 200 bp without protein-coding ability or with a weak protein-coding ability [3]. The molecular functions of lncRNA include its action as a host gene for microRNA (miRNA), preventing RNA and protein from binding to the expected targets, or acting as a molecular scaffold to guide proteins to their direct chromosomal targets [4]. MYLK-AS1 is located on chromosome 3 and is a long non-coding RNA with a length of 814 bp. It was originally reported in the Mammalian Gene Collection project of the National Institutes of Health, with the purpose of identifying putative alternative promoters for human genes, but no detailed study is yet available [5, 6]. Recently, an overall survival (OS) risk scoring system for HCC patients has been constructed based on the expression of 6 lncRNAs including MYLK-AS1 [7]. According to reports, MYLK-AS1 promotes the growth and invasion of HCC through the EGFR/HER2-ERK1/2 signaling pathway [8]. In addition, MYLK-AS1 is down-regulated in colon adenocarcinoma compared with paired adjacent normal tissues [9]. However, the specific biological function of MYLK-AS1 is still unclear, and the clinical significance of MYLK-AS1 in HCC still needs to be further determined.

The E2F transcription factor family consists of eight family members. According to in vitro experiments [10, 11], they are mainly divided into activators (E2F1–3) and inhibitors (E2F4–8) of transcription, and their role depends on the cell background, target genes and cofactors [12–14]. The classic E2F (E2F1–6) contains a DNA binding domain and forms a heterodimer with the dimerization partner protein, while the atypical family members E2F7 and E2F8 (E2F7/8) have two DNA binding domains, forming a homodimer or heterodimers and regulate the transcription without being related to the dimerization partner [15]. Recent studies showed that the activator E2Fs are dispensable for cell division, playing a key role [11, 16]. Consistent with this cell cycle-independent function of E2Fs, some studies recently demonstrated that the loss of E2f7/8 in mice causes embryo death at E11.5 days without proliferation defects [17]. In contrast, mice overexpressing E2f7/E2f8 showed a remarkable amount of apoptosis and vascular defects at E10.5. Interestingly, apoptosis was restored in these mice after the additional deletion of E2F1 or p53, but the vascular defect was also rescued, indicating that E2F7/8 regulate vascular integrity through another mechanism.

Therefore, in this study, the expression, clinical significance, function and potential mechanism of MYLK-AS1 in HCC was explored. The results revealed that MYLK-AS1 was highly expressed in HCC and promoted the proliferation, angiogenesis, metastasis and invasion of HCC cells. In addition, since miR-424-5p regulates the proliferation and apoptosis of cancer cells in HCC [18], and it can also be used as a potential biomarker in the diagnosis of liver cancer [19], the relationship among MYLK-AS1, miR-424-5p, E2F7 and VEGFR-2 signaling pathway in HCC cells was studied. Our results showed that MYLK-AS1 activated the VEGFR-2 signaling pathway through MYLK-AS1 / miR-424-5p / E2F7 axis to promote HCC progression. MYLK-AS1 and E2F7 were closely related to some malignant clinicopathological features and prognosis of HCC. Thus, the MYLK-AS1/ miR-424-5p/E2F7 signal axis might provide a novel and promising treatment strategy to combat HCC.

## Methods

### TCGA data downloading

The Cancer Genome Atlas (TCGA, www.portal.gdc.cancer.gov) was used to download the RNA-seq data of the Liver Hepatocellular carcinoma (LIHC) patients and a comprehensive analysis of these data was conducted. The screenings of the patients was performed according to the following criteria: 1) No chemotherapy or radiation performed before surgery 2) Absence of other malignancies 3) Sufficient data for analysis 4) LIHC. Fifty adjacent non-cancerous tissues and 374 tumor tissues from a total of 424 samples were analyzed. The publication guidelines issued by the TCGA (www.cancergenome.nih.gov/publications/publicationlines) were strictly followed.

### RNA-seq and analysis of the differential gene expression

The TCGA-LIHC (back dated to Oct. 1, 2017) supplied the RNA expression profiles (level 3) of the LIHC patients. The normalized transcriptomic data of the high-throughput sequencing, which included the lncRNA, miRNA and mRNA profiles, were provided by the database. Clinical data and miRNAseq were included in the corresponding RNAseq of each sample. By the use of the edgeR package [20] in R (version 3.4.3, 2017, https://www.r-project.org), the expression of miRNA, mRNA, and the differential IncRNA between the adjacent non-tumor and tumor tissues was analyzed. LncRNAs, miRNA and mRNAs with a false discovery rate (FDR) with a log fold change (FC) > 2.0 and adjusted *P* value < 0.01 were considered as differentially expressed (DElncRNA, DEmiRNAs and DEmRNAs, respectively). The volcano plot and the heatmap of DElncRNAs, DEmiRNAs, and the DEmRNAs were generated using the ‘ggplot2’ and the ‘pheatmap’ packages in R [21]. The DEmRNAs visualized through the ‘ggplot2’ and ‘clusterProfiler’ were subjected to the KEGG pathway enrichment analysis. The fold enrichment > 1.5 and *P* < 0.001 identified the affected pathways. The q value < 0.05 and the ‘survival’ value of the impact of the differentially expressed RNAs on the patient survival were analyzed.

### Construction of the ceRNA network

Only the dysregulated pool of lncRNAs and mRNAs was considered to determine the lncRNA-miRNA-mRNA interactions. Subsequently, the miRNA seed sequences serving as the shared (overlapping) binding sites for the lncRNAs and mRNAs were identified. The miRcode (www.mircode.org) predicted the lncRNA-miRNA interaction. The miRTarBase (mirtarbase.mbc.nctu.edu.tw), Targetscan (www.targetscan.org), and the miRDB (www.mirdb.org), were used to determine the miRNA targeting mRNAs. The TCGA-LIHC differential expression data and the intersecting lncRNAs and mRNAs in the miRNA predictions were selected to enhance the reliability of the ceRNA network. Cytoscape v3.5.0 [22] was used to visualize the ceRNA network interaction.

### HCC patients and tissue specimens

A total of 156 normal liver and paired HCC tissues samples from HCC patients who underwent radical surgery at the Minhang Hospital, Fudan University in China were collected between 2012 and 2019. No patient received chemotherapy or radiation therapy before surgery. The patient stage was evaluated according to the guidelines issued by the American Joint Committee on Cancer (AJCC) TNM classification (AJCC 7th edition, 2010) after the independent diagnosis by two experienced pathologists. The tissue specimens were frozen in liquid nitrogen immediately after collection and stored at − 80 °C for further RNA extraction. The Human Research Ethics Review Board of the Minhang Hospital approved the use of human cancer tissues. All patients involved in this study signed the informed consent form.

### HCC cell lines and culture conditions

The Chinese Academy of Sciences (Shanghai) Cell Bank provided the nine human cell lines used in this study, such as human embryonic kidney (HEK) 293 FT cells, human umbilical vein endothelial cells (HUVECs), and the following human HCC cell lines: human liver hepatocellular cell line Hep-G2, MHCC-97H, BEL-7402, SNU-387, MHCC-97 L, and SMMC-7721. All cell lines were cultured in RPMI-1640 (Hyclone, Logan, UT, USA) supplemented with 15% fetal bovine serum and incubated at 37 °C under 5% CO_2_.

### Animals

The Shanghai SLAC Laboratory Animal Co., Ltd. provided the four-week-old female BALB/c nude mice (4 week-old, weighing 15 g), which were kept under pathogen free conditions. Mice were housed under controlled environmental conditions of 25 °C and 12:12-h light-dark cycle, with food and water ad libitum.

### In vivo tumor formation

MHCC-97H cells transfected with MYLK-AS1 vector or empty vector were subcutaneously injected in the right axillary of the BALB/c nude mice randomly selected, in case of the MHCC-97H that were transfected. The tumor formation and the tumor volume were measured every 4 days. Four weeks after injection the mice were sacrificed and the xenograft tumors were harvested, weighed, and analyzed. The animal studies were approved by the Animal Care and Use Committee of the Fudan University.

### RNA extraction and qRT-PCR

TRIzol (Invitrogen, Carlsbad, CA, USA) was used to extract the total RNA from the cell lines, mouse tumor, and patient tissues according to the manufacturer’s guidelines. The Moloney Leukemia Virus Reverse Transcriptase Kit (Madison, Wisconsin, Promega, USA) was used for the reverse transcription reaction to generate a high-quality cDNA using 1 μg of total RNA as a template. The cDNA samples were amplified by qRT-PCR performed using the Green Mix SYBR (Promega) to measure the expression of lncRNA and mRNA, and GAPDH was used as the internal control to normalize their expression. The All-in-One™ miRNA qRT-PCR kit (GeneCopoeia, Carlsbad, CA, USA) was used to measure miRNA expression by qRT-PCR performed through the ABI 7500 fast real-time PCR system (Applied Biosystems, Waltham, Massachusetts, USA) and the U6 snRNA was used as the internal control. The FC in the relative expression was determined by the 2-^△△CT^ method and each sample was measured three times. The Table S1 of the Supplementary Document lists the primer sequences used in this study. The primers were designed using the online tool “pick primer” on NCBI website.

### In situ hybridization (ISH)

MYLK-ASI antisense oligonucleotide probes, 20 nM, for MYLK-AS1 (Exiqon Inc. Shanghai, China) were used to perform ISH. The sections were deproteinated, hydrated, and deparaffinized, and subsequently pre-hybridized in the hybridization buffer for 2 h at 55 °C in a humidified chamber. A 20 nM probe was added to the array slides covered with Nescofilm and placed in hybridization buffer (Bando Chemical Co., Kombe, Japan) in a humidified chamber, and the slides were incubated overnight at 55 °C, to perform the hybridization process. The slides were treated with the chromogen 3, 3-Diaminobenzidine (DAB) in combination with the alkaline phosphatase conjugate and anti-digoxigenin and incubated for 30 min at 37 °C to develop the brown color. The cells were counterstained with Mayer’s hematoxylin solution for 3 to 5 min and the slides were finally mounted. ISH scoring criterion: Scoring the staining in a semi-quantitative manner: 0 (no hybridization), 1+ (positive cell percentage < 25%), 2+ (positive cell percentage 25-50%) and 3+ (positive cell percentage > 50%). A score of 0 or 1+ is considered low expression, and a score of 2+ or 3+ is considered high expression. Two independent pathologists evaluated and scored each sample.

### Immunohistochemistry (IHC)

The subcutaneous tumors from nude mice were collected, embedded in paraffin, cut into 4 μm-thick sections, dewaxed, and rehydrated. The section were subjected to the retrieval of the antigen, incubated overnight with anti-CD34 (cat. 3569, CST; 1:1000) primary antibody at 4 °C, subsequently incubated with anti-HRP secondary antibody (Glostrup, Dako, Denmark) for 2 h at room temperature, and finally counterstained with Mayer’s hematoxylin solution. Two independent pathologists evaluated the IHC stain scoring. According to the estimated percentage of tumor cells that were positively stained, the score from 0 to 3 of the tumor staining intensity was the following: 0: no staining; 1: weakly stained; 2: moderately stained; 3: strongly stained. Approximately 100 cells in multiple tumor areas were analyzed to evaluate the mean staining intensity of the tumor samples. The cytoplasmic expression was determined with score ranges from 0 to 300 by the Histoscore (H-score) using the formula Histoscore = ∑(I × Pi), where Pi was the percentage of the positively stained tumor cells, and I represented the intensity [23]. Two independent pathologists blinded to the clinical results performed the scoring.

### Colony formation assay and cell proliferation

Tumor cells were seeded into a 96-well plate at a density of 100 cells/well at 24 h post-transfection and cultured for 7 days to determine cell proliferation. The kit-8 for cell counting (Dojindo, CCK-8; Tokyo, Japan) was used and the staining solution (10 μL) was added into each well every day. Cell proliferation was measured at the absorbance of 450 nm. Tumor cells were also seeded into 6-well plates at a density of 500 cells/well at 24 h post-transfection to evaluate the colony formation ability for 2 weeks. The cells were stained with 0.1% crystal violet and fixed with methanol. The colonies were counted using Quantity One (BioRad, Hercules, CA, USA). The experiments were repeated thrice and the mean values were calculated.

### RNA binding protein Immunoprecipitation (RIP) assay

The RIP assay was performed using the Magna RIP RNA-Binding Protein Immunoprecipitation reagent kit (Billerica, MA, Merck Millipore, USA). The antibody (10 μg) was added to the supernatant (10 mg) of the liver cancer cell lysate, incubated for 2 h at 4 °C under gentle agitation, and then incubated overnight. Next, protein A/G magnetic beads (40 μL) were added and incubated for 1 h at 4 °C under gentle agitation. Incubation was done with magnetic beads conjugated to IgG antibody (ab172730; 1:5000; Abcam Inc., Cambridge, MA, USA) or the Ago2 antibody (ab32381; Abcam Inc., Cambridge, MA, USA). qRT-PCR was performed to evaluate the immunoprecipitation of the RNAs.

### Lentivirus construction and cell transfection

Two types of MYLK-AS1-targeting lentiviral short-hairpin RNAs (shRNAs) were purchased from GenePharma (Shanghai, China), while the vector containing MYLK-AS1-overexpressing pLVX-IRES-ZsGreen was purchased from Takara Bio (Catalog no. 632187, CA, USA), and both were transfected into the HCC cell lines. To identify the cells with stable overexpression of MYLK-AS1 or stable knockdown, the cells were treated with 2 μg/mL puromycin for 48 h after 2 weeks post-transfection. qRT-PCR was performed to evaluate the transfection efficiency. The negative control (NC) oligonucleotides, the hsa-miR-424-5p inhibitor, and the hsa-miR-424-5p mimic were purchased from Ribobio (Guangzhou, China). The pLVX-IRES-ZsGreen-E2F7 vector used for overexpressing E2F7 was purchased from Takara Bio (CA, USA). The HCC cell lines were transfected with the above plasmids and oligonucleotides using Lipofectamine 3000 (Invitrogen) according to the manufacturer’s instructions.

### Cell migration and invasion assay

The Transwell inserts (8-μm pore size; Corning Costar, Cambridge, MA, USA) were used to perform both assays. The RPMI-1640 supplemented with 20% serum as a chemoattractant was added to the lower chamber and the non-adherent tumor cells were cultured in a serum-free RPMI-1640 medium and seeded into the top chamber and incubated for 24 to 48 h. The inserted membrane was stained with 0.1% crystal violet and fixed in methanol. The migration of the cells to the bottom of the membrane was counted and visualized under the microscope after the membrane was gently wiped on the upper surface. The cell invasion assay was performed using the same protocol with the addition of a layer of matrigel onto the Transwell membrane.

### Cell cycle and apoptosis

The cells were seeded into 6-well plates at a density of 3 × 10^5^cells/well and incubated overnight at 37 °C. The adherent cells were fixed in 70% cold ethanol and incubated overnight at − 20 °C. The cells were stained with Propidium Iodide (PI) according to the manufacturer instructions and the cell cycle distribution was evaluated by flow cytometry. As regard the evaluation of apoptosis, the cells were cultured in 2 ml culture medium, seeded into in 6-well plates at a density of 3 × 10^5^ cells/well, and incubated at 37 °C for 18–24 h. Next, cells were washed twice in cold PBS, collected, re-suspended in 100 μl binding buffer (BD Pharmingen), stained with 5 μl PI solution and 5 μl Annexin V-FITC and incubated for 20 min in the dark. Flow cytometry analysis (FACS Calibur, BD Biosciences, San Jose, CA, USA) was performed within 30 min after staining. The apoptotic rate was calculated using the following formula: Apoptotic rate = Early apoptotic (Q4) + late apoptotic (Q2).

### Fluorescence in situ hybridization (FISH)

The MHCC-97H cells were seeded into 24-well plates at a density of 3 × 10^4^ cells/well. Next, they were fixed in 4% PFA at room temperature for 15 min and permeabilized in 0.5% Triton X-100 for 15 min at 4 °C. Cells were then treated with the Control-FISH probe or the digoxigenin (DIG)-labeled MYLK-AS1 probe and incubated at 55 °C for 4 h, Next, they were washed for 5 min in 2X PBS, treated with anti-DIG secondary antibodies conjugated with horseradish peroxidase (HRP) (Jackson, West Grove, PA, USA) and counterstained with DAPI. Images were captured using the Olympus confocal laser scanning microscope.

### Luciferase reporter assay

MYLK-AS1 cDNA containing the predictive binding site of miR-424-5p was cloned into the pmirGLO Dual-Luciferase miRNA Target Expression Vector (Promega) to form the reporter vector named pmirGLO-MYLK-AS1-WT. The mutant MYLK-AS1 containing the point mutations of the miR-424-5p seed region binding site was specifically synthesized and inserted into the abovementioned vector, which was named pmirGLO-MYLK-AS1-Mut. HEK-293FT cells were cultured into 96-well plates and co-transfected with pmirGLO-MYLK-AS1–3′-UTR vectors including wild-type or mutant fragments and miR-424-5p mimic, and the pmirGLO vector was used as the NC. To confirm the direct interaction of miR-424-5p and E2F7, wild-type and mutant E2F7 3′-UTR fragments were amplified by qRT-PCR and cloned into the pmirGLO vector (Promega) using the one-step directed cloning kit (Novoprotein, Shanghai, China); the resultant vectors were termed E2F7-WT and E2F7-Mut, respectively. The miR-424-5p mimic was co-transfected with E2F7-WT or E2F7-Mut vector into HEK-293FT cells using Lipofectamine 3000 (Invitrogen). The luciferase assay was performed at 48 h post-transfection using the Dual Luciferase Reporter Assay System (Promega) according to the manufacturer’s instructions. Relative firefly luciferase activity was normalized to Renilla luciferase activity as a control for transfection efficiency. The primers used for vector construction are provided in the Additional file 1: Table S1. These results are presented as mean ± standard deviation (SD) of three replicates.

### Immunoblotting

Hep-G2 and MHCC-97H cells (both used as control, shRNA-NC, shMYLK-AS1, NC, and MYLK-AS1 overexpression) were seeded into 6-well plates at a density of 3 × 10^5^ cells/well. Total protein was extracted using the RIPA buffer (cat.9806, CST) supplemented with the kinase inhibitors and protease. The nucleic proteins and total cytoplasmic proteins were extracted using the PROTTOT-1KT ProteoPrep kit (Sigma). Separating equal quantities an amount of 30 μg protein was subjected to SDS-PAGE electrophoresis and transferred onto a PVDF membrane. The membrane was subsequently blocked with 5% BSA in PBS, and incubated with the primary antibodies and their corresponding secondary antibodies. Proteins were visualized using the Western Lightning Plus-ECL (PerkinElmer, Waltham, Massachusetts, USA). The monoclonal antibodies used were the following: anti-PLC-λ (cat.5690, CST; 1:1000), anti-p-VEGFR-2(cat.2478, CST; 1:1000), anti-VEGFR-2 (cat.9698, CST; 1:1000), anti-E2F7 (cat.ab56022, Abcam; 1:1000), anti-p-P38 (cat.4511, CST; 1:1000), anti-FAK (cat.71433, CST; 1:1000), anti-p-PLC-λ (cat.8713, CST; 1:1000), anti-p-Erk1/2 (cat.4370, CST; 1:1000), anti-P38 (cat.8690, CST; 1:1000), anti-Erk1/2 (cat.4695, CST; 1:1000), anti-p-Src (cat.6943, CST; 1:1000), anti-Akt (cat.4685, CST; 1:1000), anti-p-FAK (cat.8556, CST; 1:1000), anti-Src (cat.2109, CST; 1:1000), anti-p-Akt (cat.4060, CST; 1:1000), anti-mouse IgG (cat.7076, CST; 1:3000), anti-GAPDH (cat.51332, CST; 1:1000) used as the loading control. All these primary antibodies were rabbit anti-human except for the GAPDH that was mouse anti-human, and the HRP-conjugated goat anti-rabbit (cat.7074, CST; 1:3000) was used as a secondary antibody, except for the GAPDH with which an anti-mouse IgG, HRP-linked secondary antibody (cat.7076, CST; 1:3000) was used.

### Statistical analysis

Statistical analysis was performed using SPSS 23.0 (SPSS, Chicago, IL, USA) or GraphPad Prism 7 (GraphPad Prism, Inc., La Jolla, CA, USA). Data were expressed as mean ± SD of three independent experiments. Two or three group comparison was carried out using Student’s *t*-test or ANOVA, respectively. The relationship between the clinicopathological variables and MYLK-AS1 expression was determined using the chi-square test. The survival was estimated by the Kaplan-Meier curve, while the difference between survival curves was analyzed using the log-rank test. The prognostic value of MYLK-AS1 for hepatocellular carcinoma was evaluated by the receiver operating characteristic (ROC) curve, which was drawn using the ‘pROC’ package in R. A value of *P* < 0.05 was considered statistically significant.

## Results

### TCGA data analysis

**Fig. 1:**
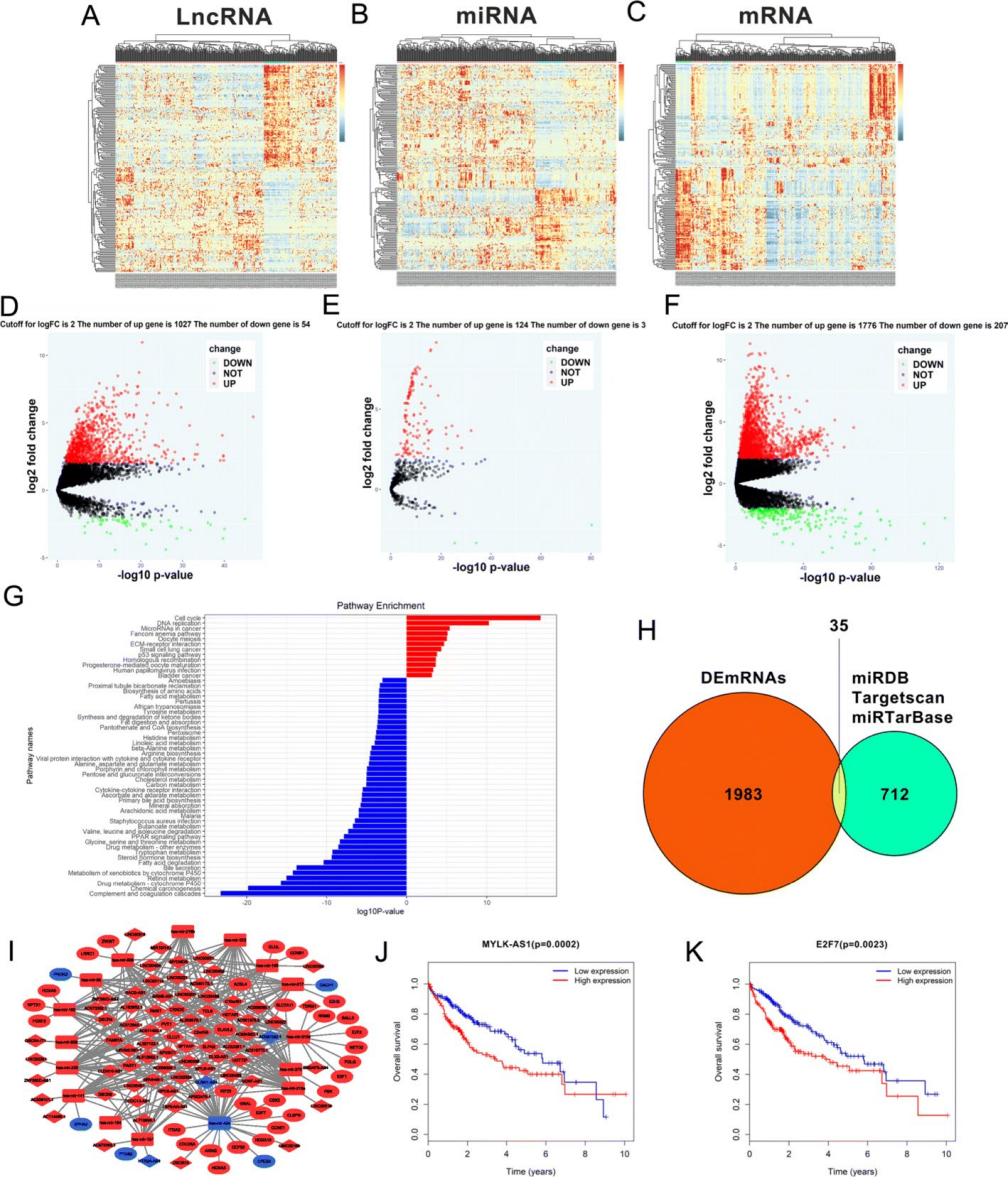
RNA-seq data analysis of HCC in TCGA database. **a-c** Clustered heat maps of the differentially expressed RNAs in HCC tissues and adjacent non-tumor liver tissues. Rows represent RNAs, whereas columns represent HCC tissues and adjacent non-tumor liver tissues. Differentially expressed lncRNAs, miRNAs, and mRNAs in HCC tissues compared to adjacent non-tumor liver tissues were filtered by standard of log2FC > 2 and FDR < 0.01. FC: folds change; FDR: false discovery rate. **d-f** Volcano plots visualizing and assessing the variation of (D) long non-coding RNAs, (E) microRNAs, and (F) mRNAs expression between HCC tissues and adjacent non-tumor liver tissues. The values of the X-axis the indicate 10log *p* value and the Y-axis indicate the log2 fold change of the group. **g** Enriched KEGG pathways for differentially expressed mRNAs (the bar plot shows the enrichment scores of the significantly enriched KEGG pathways). KEGG, Kyoto Encyclopedia of Genes and Genomes. **h** Identification of 712 changed targeted mRNAs among the 127 DEmiRNAs from the three public profile datasets (miRDB, Targetscan and miRTarBase). The cross areas revealed that the number of commonly changed mRNAs between DEmRNAs and target mRNAs was 35, which included E2F7. **i** The lncRNA-miRNA-mRNA ceRNA network. Blue squares, downregulated miRNAs; blue circles, downregulated mRNAs; blue diamonds, downregulated lncRNAs. Red squares, upregulated miRNAs; red circles, upregulated mRNAs; red diamonds, upregulated lncRNAs. **j-k** Kaplan-Meier survival curves for MYLK-AS1 and E2F7 associated with OS

A total of 1081 lncRNAs (1027/95.0% upregulated and 54/5.0% downregulated), 127 miRNAs (124/97.6% upregulated and 3/2.4% downregulated), and 1983 DEmRNAs (1776/89.6% upregulated and 207/10.4% downregulated), were identified on 50 adjacent non-tumor samples and 374 HCC samples obtained from TCGA after filtering with logFC > 2 and *P* < 0.01 (Fig. 1a-f). The KEGG pathway enrichment analysis performed on these 1983 DEmRNAs revealed that the down- and up-regulated DEmRNAs were most enriched in ‘Complement and Coagulation Cascades’ and ‘Cell cycle’, respectively (Fig. 1g). Moreover, 43 pathways correlated with the downregulated DEmRNAs and 12 pathways associated with the upregulated DEmRNAs were identified. A total of 712 target mRNAs were identified and 127 DEmiRNAs were predicted using the miRTarBase and the miRDB, Targetscan. Thirty five intersecting mRNAs interacting with the 16 DEmiRNAs were selected between the 1983 DEmRNAs and the 712 predicted target mRNAs (Fig. 1h) to construct the ceRNA network. A lncRNA-miRNA-mRNA ceRNA network involving 35 DEmRNAs, 16 DEmiRNAs, and 74 DElncRNAs was constructed using Cytoscape 3.5.0 to understand the mechanisms used by the lncRNAs to bind the miRNAs and regulate the mRNA expression in HCC (Fig. 1i). Some mRNAs in the ceRNA network were previously known as linked with cancer including the E2F Transcription Factor 7 (E2F7), Solute Carrier Family 7 Member 11 (SLC7A11), Kinesin Family Member 23 (KIF23), Chromobox 2 (CBX2), Enhancer of Zeste 2 Polycomb Repressive Complex 2 Subunit (EZH2), and Cell Division Cycle 25A (CDC25A). Subsequently, an analysis of the OS of HCC patients and the association between the differentially expressed genes in the ceRNA network revealed that 19 of the 35 DEmRNAs and 14 of the 74 DElncRNAs, including E2F7 and MYLK-AS1, were significantly associated with OS (Fig. 1j, k, and data not shown). Our data suggested that the MYLK-AS1/miR-424-5p/E2F7 axis played a potentially adverse role in the prognosis of HCC patients when miR-424-5p was low expressed in cancer tissues and this axis was the only ceRNA regulatory one that indicated a negative association with OS at both its lncRNA and mRNA ends.

### MYLK-AS1 overexpression in the HCC tissues

**Fig. 2:**
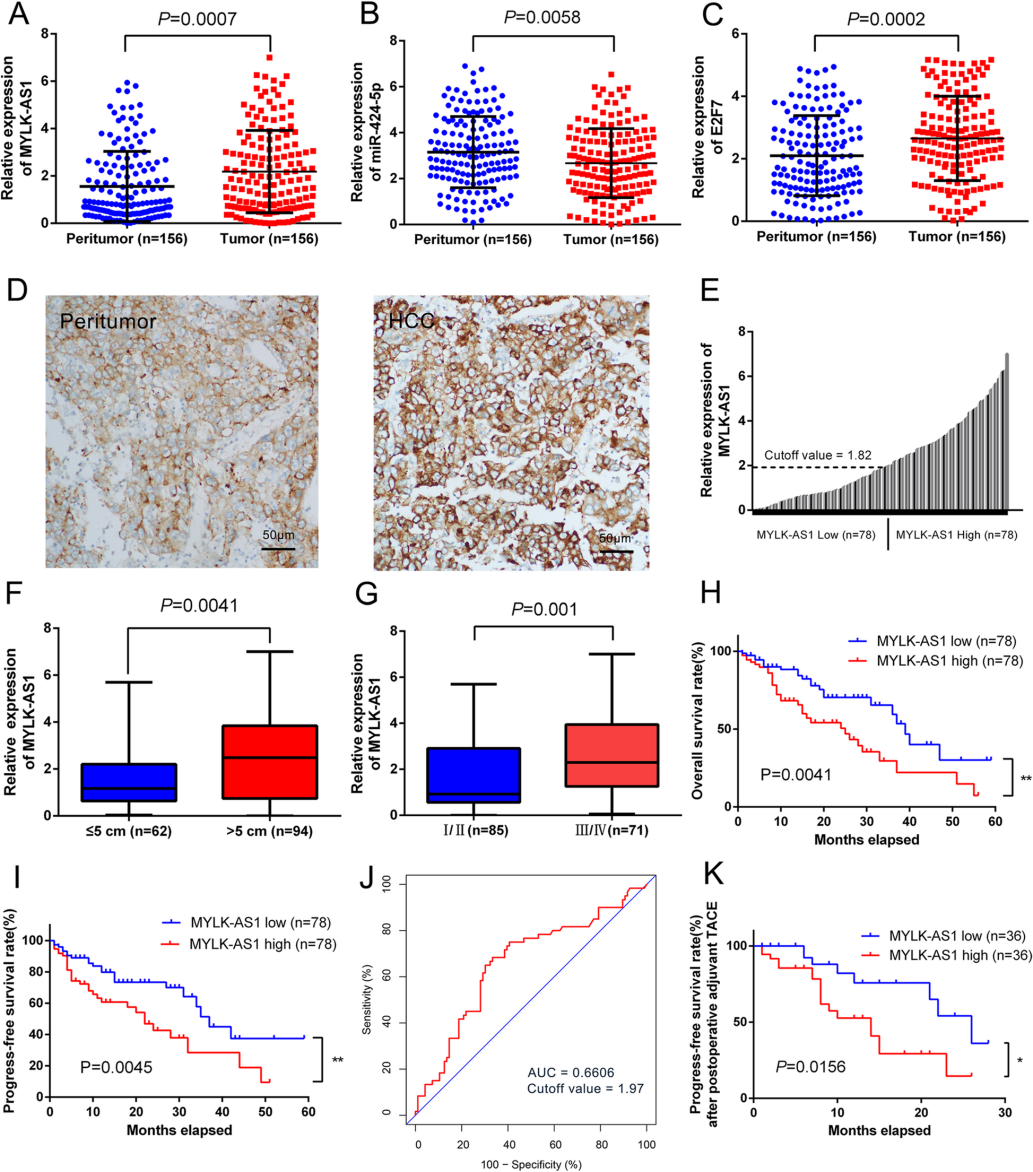
MYLK-AS1 and E2F7 overexpression is positively correlated with HCC progression and poor prognosis. **a-c** Relative expression of MYLK-AS1, miR-424-5p and E2F7 detected by qRT-PCR in 156 paired HCC cancer tissues and matched normal liver tissues. Results are presented as the relative expression (compare to internal control, the 2-△△CT method) in tumor tissues and peritumoral tissues. **d** MYLK-AS1 expression in peritumoral tissues and HCC tissues by ISH. **e** Relative MYLK-AS1 expression by qRT-PCR in 156 HCC tissues. Relative MYLK-AS1 expression presented as the relative expression (compare to internal control, the 2-△△CT method) in tumor tissues and the matched normal tissues. HCC patients were divided into high (*n* = 78) and low (*n* = 78) group according to the median value (0.50). **f-g** Relative MYLK-AS1 expression in HCC with different size and stage. Results were presented as the relative expression (compare to internal control, the 2-△△CT method) in tumor tissues and normal tissues. **h-i** Kaplan-Meier plots of the OS and PFS of HCC patients with high (*n* = 78) and low (*n* = 78) MYLK-AS1 expression. Data are presented as mean ± SD. *P < 0.05, **P < 0.01, and ***P < 0.001. **j** ROC analysis of the performance of MYLK-AS1 expression of 1-year OS in all patients. K Kaplan-Meier plots of the PFS of HCC patients after postoperative adjuvant TACE therapy with high (*n* = 36) and low (*n* = 36) MYLK-AS1 expression. Data are presented as mean ± SD. *P < 0.05, **P < 0.01, and ***P < 0.001

The HCC tissue from patients showed an upregulated MYLK-AS1 expression (*P* = 0.0007), downregulated miR424-5p expression (*P* = 0.0058), and upregulated E2F7 expression (*P* = 0.0002) when compared with the healthy tissue counterpart (Fig. 2a-c). ISH experiments confirmed that the expression of MYLK-AS1 in HCC tissues was much higher than that of the adjacent tissues and further confirmed that MYLK-AS1 was mainly located in the cytoplasm (Fig. 2d). The median score 1.82 of the relative MYLK-AS1 expression was defined as the cutoff value to evaluate the prognostic value of MYLK-AS1 overexpression (Fig. 2e). The stratification of the 156 HCC patients into two groups according to the median MYLK-AS1 expression and the correlation between the clinicopathological characteristics and MYLK-AS1 expression revealed that the TACE treatment, vascular invasion, tumor differentiation, tumor size, and tumor stage were significantly associated with MYLK-AS1 expression (Table 1). Furthermore, MYLK-AS1 expression in tumors with a size >5 cm was significantly higher than that in the tumors with a size ≤5 cm, and MYLK-AS1 expression at the tumor III/IV stage was significantly higher than that at the tumor I/II stage (Fig. 2f, g). Nonetheless, no significant difference in MYLK-AS1 expression among the subgroups of TACE treatment, vascular invasion, and tumor differentiation was observed (Additional file 2: Fig. S1A-C). The upregulation of MYLK-AS1 was often observed in HCC (on both the TCGA database and in the patients of our hospital), thus, its association with metastasis and HCC progression could not be excluded.

**Table 1:** Relationship between MYLK-AS1 and clinicopathological parameters in 156 HCC patients

Clinical variables	Number of cases	MYLK-AS1 expression		*P* value
		Low *N* = 78	High *N* = 78	
Age (years)				
> 50	26	15	11	0.390
≤ 50	130	63	67	
Gender				
Male	103	48	55	0.237
Female	53	30	23	
Tumor number				
Single	125	65	60	0.316
Multiple	31	13	18	
Etiology				
viral	122	58	64	
Non-viral	34	20	14	0.245
Serum AFP (ng/ml)				
≤ 200	75	33	42	0.149
> 200	81	45	36	
Tumor stage				
I/II	85	52	33	0.002
III/IV	71	26	45	
Tumor size (cm)				
≤ 5	62	41	21	0.001
> 5	94	37	57	
Tumor differentiation				
Well	58	37	21	0.018
Moderate	49	23	26	
Poor	49	18	31	
Vascular invasion				
Yes	50	19	31	0.040
No	106	59	47	
TACE treatment				
Yes	72	28	44	0.010
No	84	50	34	

### Positive correlation of MYLK-AS1 overexpression with poor prognosis and HCC progression

A study of the correlation of the progression-free survival (PFS) of HCC patients and MYLK-AS1 expression with the OS was done, revealing that patients with tumor tissue with high MYLK-AS1 expression displayed a significantly shorter OS (30.6% vs. 59.3%, *P* = 0.0041) and PFS (27.2% vs. 48.9%, *P* = 0.0045), in comparison with patients with low MYLK-AS1 expression, according to the Kaplan-Meier analysis (Fig. 2h, i). As regard the ROC curve of MYLK-AS1 for the 1-year overall survival of HCC patients, the AUC was 0.6606 (Fig. 2j), and the ROC curve of MYLK-AS1 for tumor size, tumor stage, tumor differentiation, and vascular invasion resulted in an AUC of 0.6438, 0.6725, 0.5785 and 0.5600, respectively (Additional file 2: Fig. S1D). Adjuvant TACE is often used in the prevention of tumor recurrence, thus, PFS after adjuvant postoperative TACE, was determined, revealing that PFS depended from the response to the adjuvant TACE therapy. In addition, MYLK-AS1 expression was also significantly correlated with the TACE treatment (Table 1). The median score 2.16 of the relative MYLK-AS1 expression was used as the cutoff value to divide the HCC patients into low and high-expression group after the adjuvant TACE (Additional file 2: Fig. S1E). The patients with high MYLK-AS1 expression responded poorly to the adjuvant TACE therapy, as confirmed by the Kaplan-Meier analysis revealing a lower PFS than the PFS of patients with a low MYLK-AS1 expression (Fig. 2k). All these results suggested that MYLK-AS1 could be considered a potential prognostic factor in HCC patients.

### MYLK-AS1 as a ceRNA of miR-424-5p in HCC

**Fig. 3:**
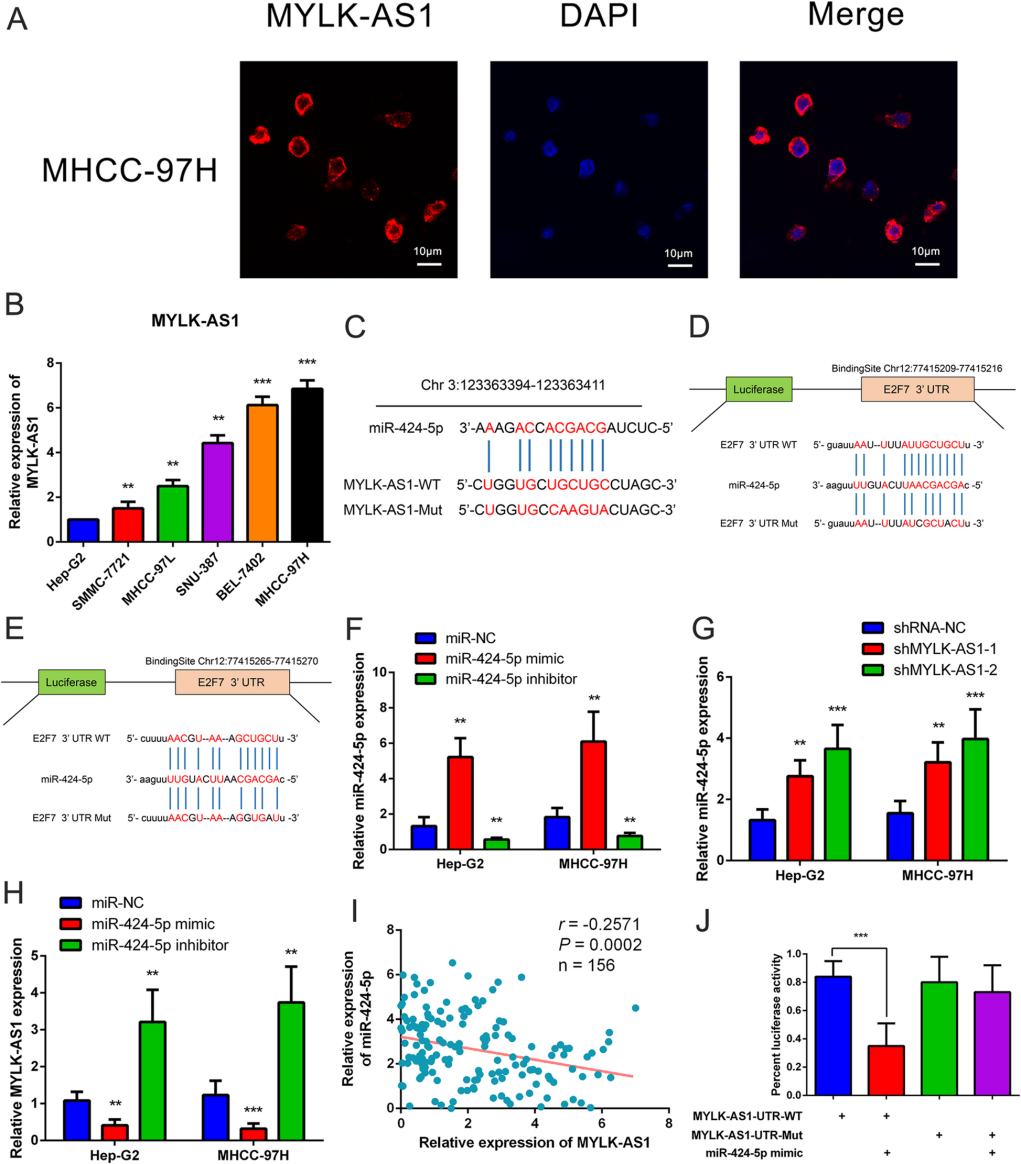
MYLK-AS1 is a ceRNA of miR-424-5p in HCC. **a** MYLK-AS1 (red) detection in MHCC-97H cells by FISH. The nucleus was counterstained with DAPI (blue). **b** Relative MYLK-AS1 expression in six HCC cell lines by qRT-PCR. **c** Schematic representation of the predicted binding site for miR-424-5p in MYLK-AS1 by the online database Mircode Predicted algorithm. The mutated site in the 3′-UTR of MYLK-AS1 is shown as MYLK-AS1-Mut. The numbers indicate the positions of the nucleotides in the reference wild-type sequence of MYLK-AS1 (Ensembl version: ENSG00000239523). **d-e** Schematic representation of the predicted miR-424-5p target site within the 3′-UTR of E2F7. The predicted target site for miR-424-5p is located at the proximal portion of the E2F7 3′-UTR. Two nucleotides complementary to the seed sequence of miR-424-5p were mutated in the E2F7 mutant plasmid. The number indicates the position of the nucleotides in the reference wild-type sequence of E2F7 (NM_203394.3). **f** Relative miR-424-5p expression in Hep-G2 and MHCC-97H cells transfected with miR-424-5p mimic or inhibitor. **g** Relative miR-424-5p expression in Hep-G2 and MHCC-97H cells after transfection with shMYLK-AS1 or scramble sequence. **h** Relative MYLK-AS1 expression in Hep-G2 and MHCC-97H cells transfected with miR-424-5p mimic or inhibitor. **i** Correlation analysis between MYLK-AS1 and miR-424-5p expression in 156 HCC tissues. **j** Relative luciferase activity of the wild type (WT) and mutated (Mut) MYLK-AS1 reporter plasmid in human embryonic kidney (HEK) 293FT cells transfected with miR-424-5p mimic. Results are expressed as mean ± SD. n.s, not significant, *P < 0.05, **P < 0.01, and ***P < 0.001

The MYLK-AS1 transcript was primarily localized in the cytoplasm as illustrated in Fig. 3a. The negative regulation of the target mRNAs by the sponging miRNAs are attenuated by the cytoplasmic IncRNAs acting as ceRNAs [24]. The Hep-G2 cells and the MHCC-97H were used because they had the lowest and the highest MYLK-AS1 expression respectively, (Fig. 3b). The involvement of E2F7, miR-424-5p, and MYLK-AS1 in the regulatory ceRNA network was hypothesized according to the clinical sample results and the TCGA data analysis. The mircode and starbase methods were used to predict the binding site between miR-424-5p and MYLK-AS1 (Fig. 3c) and between E2F7 and miR-424-5p (Fig. 3d, e). The MHCC-97H and Hep-G2 cells were transfected with an inhibitor and a miR-424-5p mimic, respectively (Fig. 3f) to downregulate or upregulate miR-424-5p expression to confirm our hypothesis. miR-424-5p was significantly upregulated by the knockdown of MYLK-AS1 (Fig. 3g), but the downregulation and upregulation of miR-424-5p respectively, increased and decreased MYLK-AS1 expression (Fig. 3h). Accordingly, miR-424-5p expression was negatively correlated with MYLK-AS1 expression in the HCC tissues (Fig. 3i). HEK293FT cells with a mutated MYLK-AS1 (mutated MYLK-AS1-Mut) or a wild-type MYLK-AS1 (MYLK-AS1-WT) binding site into a dual-luciferase reporter were used to evaluate the potential interaction between miR-424-5p and MYLK-AS1. The miR-424-5p was a direct target of the MYLK-AS1 as confirmed by the luciferase activity of MYLK-AS1-Mut remaining unchanged, despite significantly decreased luciferase activity of MYLK-AS1-WT was observed in HEK293FT cells after co-transfection with miR-424-5p mimic, as shown in Fig. 3j. Comprehensively, our data confirmed that miR-424-5p was regulated by MYLK-AS1 as a ceRNA.

### MYLK-AS1 promotes angiogenesis in the HCC tissue by targeting miR-424-5p/E2F7

**Fig. 4:**
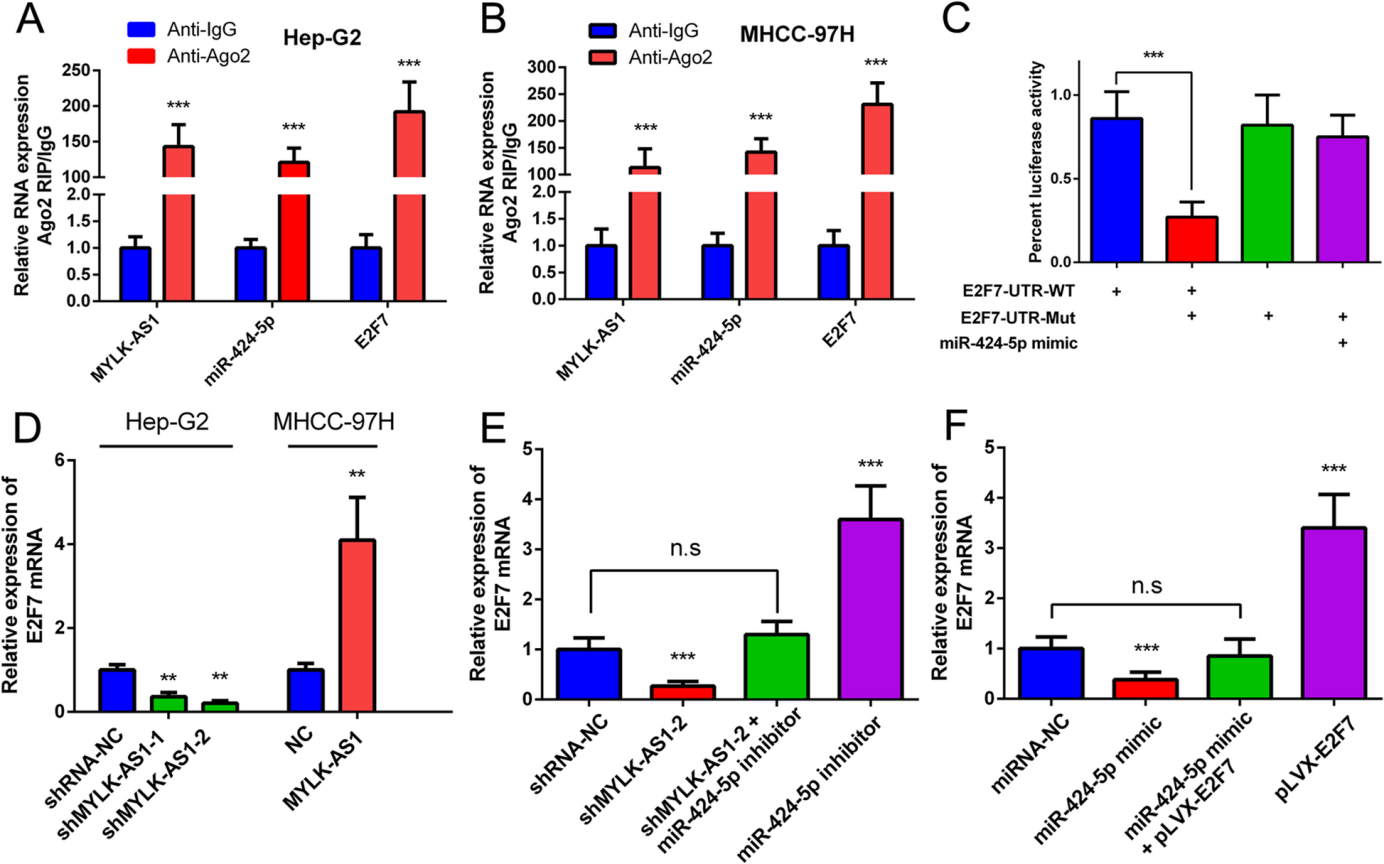
MYLK-AS1 up-regulates E2F7 expression by competitively binding miR-424-5p in HCC. **a-b** Binding ability of MYLK-AS1, miR-424-5p, and E2F7 to anti-Ago2 in Hep-G2 and MHCC-97H (anti-igG was used as control) by RIP assay. **c** HEK-293FT cells co-transfected with wide type (WT) or mutated (Mut) E2F7 3′-UTR reporter vector and miR-424-5p mimic by luciferase reporter assay. **d** Relative E2F7 mRNA expression in Hep-G2 cells after MYLK-AS1 knockdown or in MHCC-97H cells after MYLK-AS1 overexpression. **e** E2F7 mRNA expression in MHCC-97H cells after reducing the expression of MYLK-AS1 and/or inhibition of miR-424-5p by qRT-PCR. **f** E2F7 mRNA expression in MHCC-97H cells following the ectopic expression of miR-424-5p and/or pLVX-E2F7 expression vector lacking the 3′-UTR by qRT-PCR. Results are expressed as mean ± SD. n.s, not significant, *P < 0.05, **P < 0.01, and ***P < 0.001

miRNAs regulate protein expression through the degradation of mRNAs or the inhibition of the mRNA translation [25]. miR-424-5p binds 12 mRNAs as revealed by the RNAseq analysis and binding site predictions from starbase (Fig. 1i). The upregulation of 6 mRNAs (CDC25A, E2F7, CCNE1, CEP55, CBX2, and CLSPN) was negatively correlated with patient survival in the 12 target mRNAs. Nonetheless, the analysis of the results of the preliminary experiments revealed that miR-424-5p and E2F7 had the strongest targeted binding ability. E2F7, MYLK-AS1, and miR-424-5p were significantly enriched in the anti-Ago2 group compared to the (anti-igG) negative control group according to the RIP experiment (Fig. 4a and b). miR-424-5p complementary bind the 3′-UTR of E2F7, as demonstrated by the luciferase assay (Fig. 4c). MYLK-AS1 overexpression or knockdown upregulated or downregulated E2F7 mRNA expression are shown in Fig. 4d. The inhibitory effect of MYLK-AS1 on E2F7 expression was partially but consistently reversed by the treatment with miR-424-5p inhibitor (Fig. 4e). In addition, the MHCC-97H cells co-transfected with miR-424-5p mimic and pLVX-E2F7 showed a partially restored E2F7 expression compared with its expression in the cells transfected with miR-424-5p mimic only (Fig. 4f).

### HCC cell invasion, migration, and proliferation regulated by MYLK-AS1 expression

**Fig. 5:**
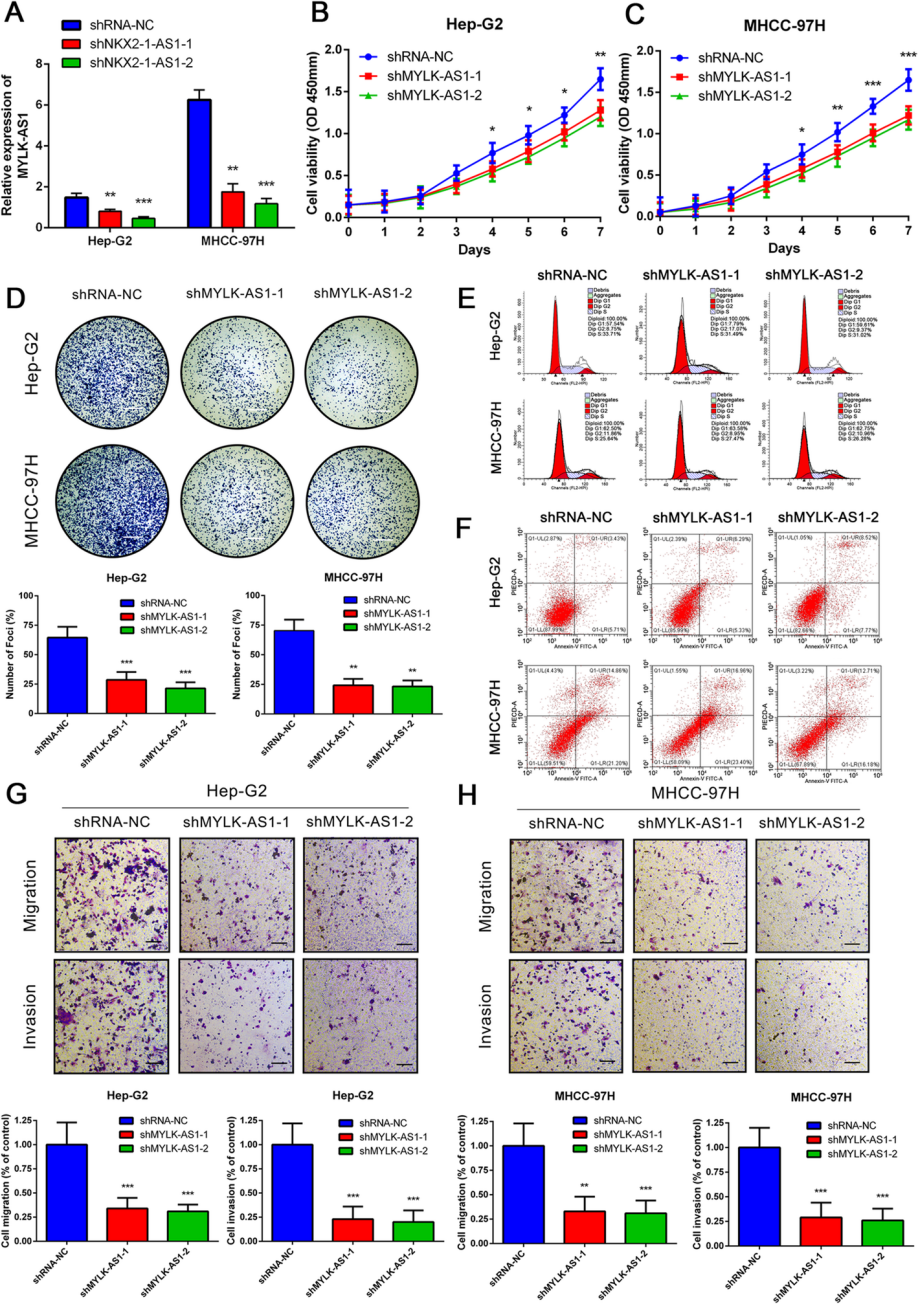
Reduced expression of MYLK-AS1 decreases the proliferation, migration and invasion of HCC cells. **a** Relative MYLK-AS1 expression in Hep-G2 and MHCC-97H cells transfected with two independent shRNAs targeting MYLK-AS1 by qRT-PCR. **b-c** Hep-G2 and MHCC-97H cell proliferation after knockdown of MYLK-AS1 by CCK-8 assay. **d-h** Representative results of the colony formation (scale bar = 500 μm), cell cycle assay, apoptosis assays, and transwell assay (scale bar = 100 μm), in Hep-G2 and MHCC-97H cells after shMYLK-AS1–1 or shMYLK-AS1–2 transfection. Results are expressed as mean ± SD. *P < 0.05, **P < 0.01, and ***P < 0.001

**Fig. 6:**
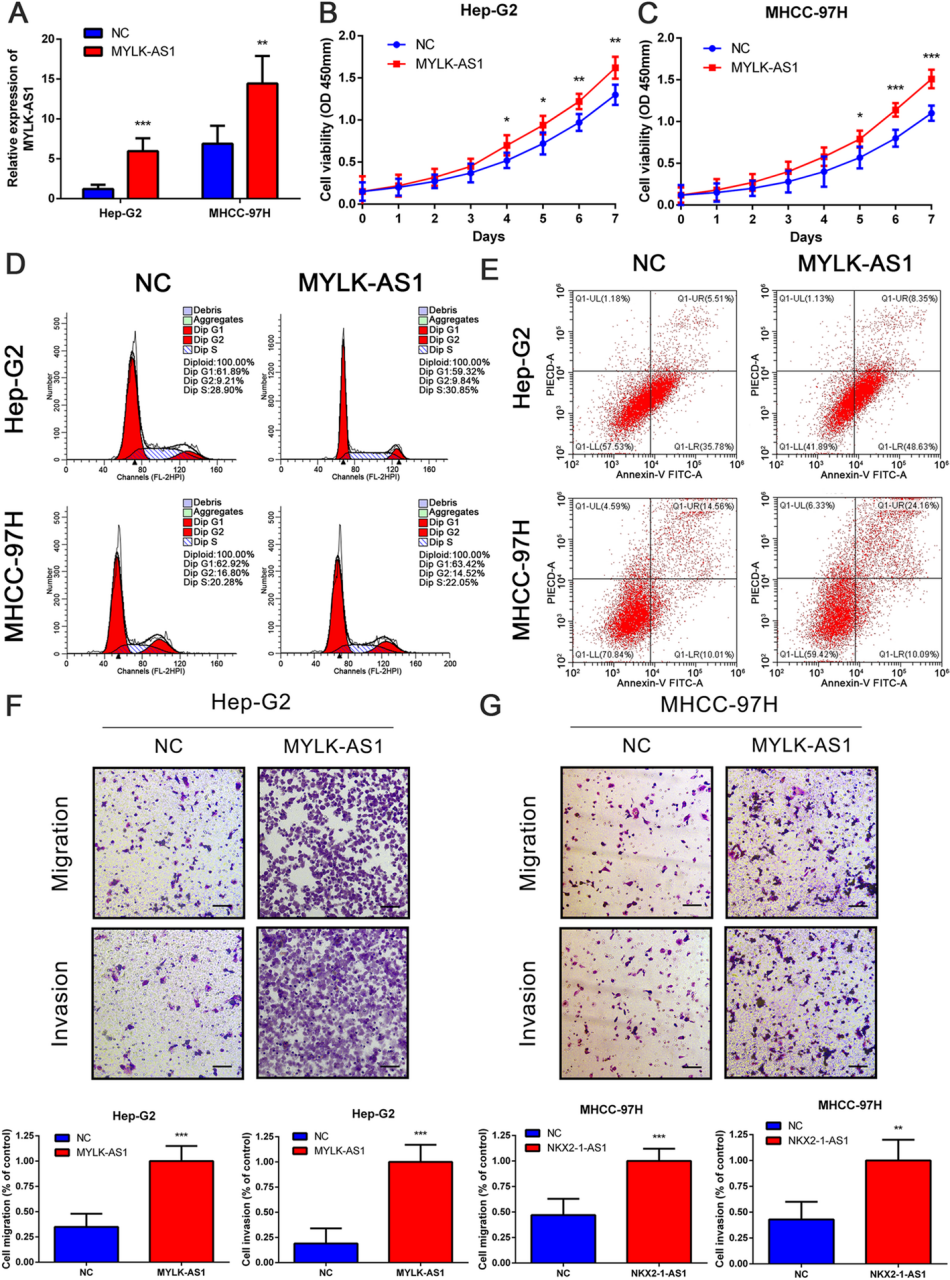
Overexpression of MYLK-AS1 promotes the proliferation, migration and invasion of HCC cells. **a** Relative MYLK-AS1 expression in Hep-G2 and MHCC-97H cells after MYLK-AS1 overexpression by qRT-PCR. **b** Proliferation of Hep-G2 and MHCC-97H cells after MYLK-AS1 overexpression by CCK-8 assay. **d-e** Representative results of the cell cycle and apoptosis assay of Hep-G2 and MHCC-97H cells after MYLK-AS1 overexpression. **f-g** Migration and invasion of Hep-G2 and MHCC-97H cells after MYLK-AS1 overexpression by transwell assay (scale bar = 100 μm). Results are expressed as mean ± SD. *P < 0.05, **P < 0.01, and ***P < 0.001

MYLK-AS1 was successfully silenced (Fig. 5a) and resulted in an inhibition of the HCC cell growth and colony formation (Fig. 5b-d), which was reversed by the overexpression of MYLK-AS1 (Fig. 6a-c). However, no significant changes were observed in the apoptosis and cell cycle rates of the HCC cells after MYLK-AS1 knockdown (Fig. 5e, f) or its overexpression (Fig. 6d, e). Besides, MYLK-AS1 overexpression (Fig. 6f, g) reversed the invasion and migration ability of the HCC cells (Fig. 5g, h), which was suppressed by the MYLK-AS1 knockdown. These results suggested that the MYLK-AS1 expression had no impact on the HCC apoptosis and the percentages of the cell cycle phases, although it regulated HCC cell invasion, growth, and migration in vitro.

### In vivo regulation of the HCC cell proliferation and angiogenesis by MYLK-AS1

**Fig. 7:**
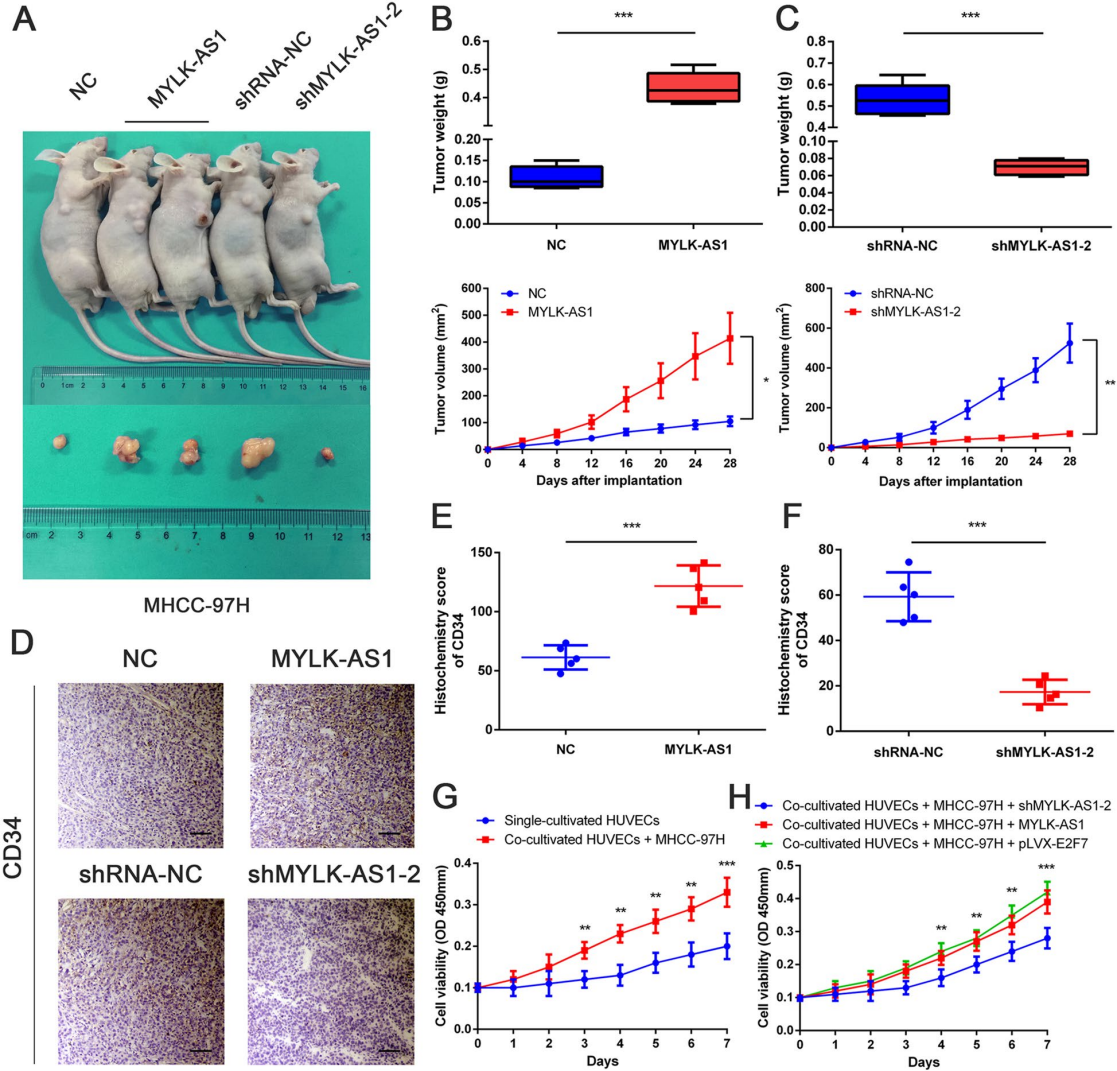
MYLK-AS1 regulates HCC cell proliferation and angiogenesis in vivo and in vitro. **a** Injection in the right armpit of MHCC-97H cells transfected with empty vector or MYLK-AS1 expression vector and shMYLK-AS1-NC or shMYLK-AS1–2 in the upper panel. Representative images of xenograft tumors are shown in the bottom panel. **b-c** Tumor weight and volume of the xenograft in MYLK-AS1 overexpression groups and control group or MYLK-AS1 knockdown group and control group. **d** Representative IHC staining results of CD34 in corresponding xenografts (scale bar = 50 μm). **e-f** Statistical analysis of the H-score of CD34 in the corresponding xenografts. Results are presented as mean ± SD from three independent experiments. **g** Cell proliferation of HUVECs cells cultured alone and co-cultured with MHCC-97H cells by CCK-8. **h** MYLK-AS1 knocked down or overexpressed or E2F7 overexpressed MHCC-97H cells co-cultured with HUVECs cells and consequent HUVEC proliferation by CCK-8. Results are expressed as mean ± SD. *P < 0.05, **P < 0.01, and ***P < 0.001

MHCC-97H cells stably expressing MYLK-AS1 were subcutaneously injected into female nude mice to evaluate the effect of MYLK-AS1 on HCC tissues in vivo (Fig. 7a). The xenograft tumors were larger and heavier in the MYLK-AS1 group as compared to the NC group (Fig. 7b). MHCC-97H cells transfected with MYLK-AS1 shRNA were also subcutaneously injected into the female nude mice, but in this case, the tumors were smaller and lighter in the shMYLK-AS1 group compared to the shRNA-NC group (Fig. 7c). The necropsy revealed that the tumors were paler in the shMYLK-AS1 group compared to those in the shRMA-NC group, suggesting that MYLK-AS1 expression could be associated with the angiogenesis of the HCC tissue, thereby impacting the growth of the tumor. The enhanced CD34 expression in tumors overexpressing MYLK-AS1 could be reversed by MYLK-AS1 knockdown, as revealed by the IHC staining (Fig. 7d-f). Hence, our in vivo results demonstrated that MYLK-AS1 was positively correlated with increased angiogenesis and tumor growth. HUVECs co-cultured with MHCC-97H displayed enhanced proliferation when compared with the proliferation of HUVECs alone (Fig. 7g). Moreover, HUVECs co-cultured with MHCC-97H overexpressing both E2F7 and MYLK-AS1 showed an increased proliferation than that of HUVECs co-cultured with MYLK-AS1-silenced MHCC-97H cells (Fig. 7h). Overall, these findings demonstrated the promoting effect of MYLK-AS1 in HCC angiogenesis depending on the miR-424-5p/E2F7 axis.

### Role of VEGFR-2 signaling pathway on MYLK-AS1/miR-424-5p/E2F7 axis regulating HCC metastasis, invasion, and angiogenesis

**Fig. 8:**
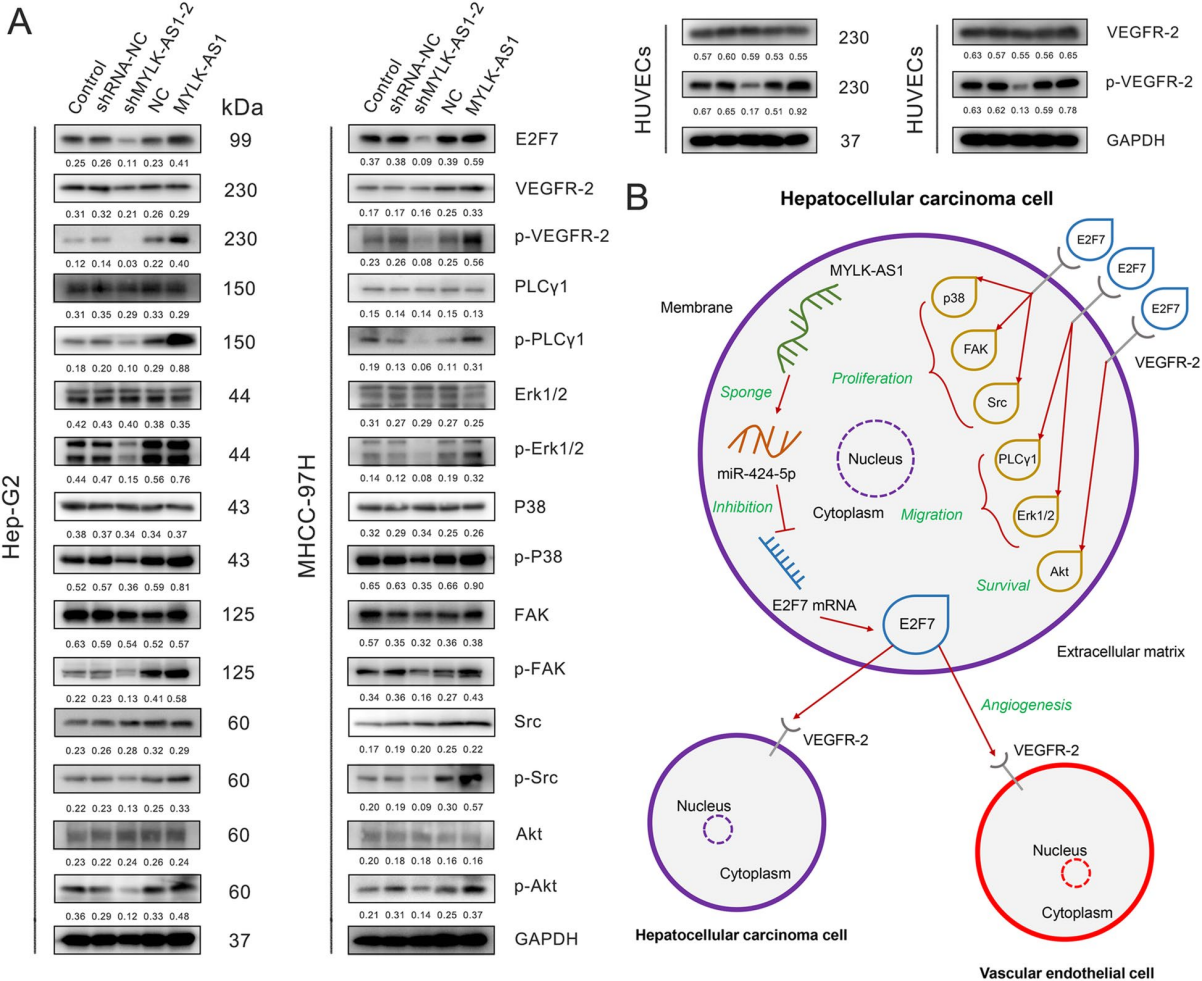
MYLK-AS1/miR-424-5p/E2F7 axis positively regulates HCC metastasis, invasion, and angiogenesis via the VEGFR-2 signaling pathway. **a** E2F7, VEGFR-2, p-VEGFR-2, PLC-λ, p-PLC-λ, Erk1/2, p-Erk1/2, P38, p-P38, FAK, p-FAK, Src, p-Src, Akt, and p-Akt protein expression in Hep-G2 or MHCC-97H cells divided in the following groups: Control, shRNA-NC, shMYLK-AS1-2, NC, and MYLK-AS1. NC represents a blank plasmid control, and MYLK-AS1 is a group overexpressing this lncRNA. The numbers represent the quantification of the relative protein amount. GAPDH was used as the loading control. VEGFR-2 and p-VEGFR-2 protein expression detected after 7-day-co-culture of HUVECs with Hep-G2 or MHCC-97H cells divided in the same groups as above. GAPDH was used as the loading control. **b** Schematic diagram of the regulatory mechanism of MYLK-AS1/miR-424-5p/E2F7 axis in the promotion of HCC proliferation, metastasis and angiogenesis

E2F7 has a significant role in tissue angiogenesis, thus, its role in HCC cells was evaluated on the canonical VEGFR-2 signaling pathway through the MYLK-AS1/miR-424-5p axis. MYLK-AS1 knockdown and overexpression did not exert any significant impact on the expression of the proteins survival-related Akt, migration-related p38, FAK, Src, proliferation-related PLCγ1, Erk1/2, and the angiogenesis-related VEGFR-2. However, the knockdown of MYLK-AS1 reduced the protein phosphorylation in all the proteins aforementioned, while its overexpression resulted in an increased protein phosphorylation, confirming the active role of MYLK-AS1 in the VEGFR-2 signaling regulation. The HUVEC cells showed also similar results when co-cultured with HCC cells with MYLK-AS1 knockdown or overexpression (Fig. 8a). These findings comprehensively indicated the dependence of the HCC cell survival, migration, proliferation and angiogenesis on the VEGFR-2 pathway being regulated by the MYLK-AS1/miR-424-5p/E2F7 axis.

## Discussion

The presence of dysregulated lncRNA expression was recently noticed in gastrointestinal cancers and HCC [26]. It was also previously demonstrated that MYLK-AS1 promotes the growth of the HCC and invasion through the EGFR/HER2-ERK1/2 signaling pathway [8]. Besides, MYLK-AS1 association with the transcription factor MYLK was known to contribute to the HCC progression by regulating the cytoskeleton to enhance the epithelial-mesenchymal transition [27]. The significant upregulation of MYLK-AS1 in HCC was confirmed in this study, promoting cancer cell proliferation and angiogenesis, both and in vivo and in vitro*.* A positive correlation of MYLK-AS1 with the invasion potential and metastasis in vitro was also demonstrated. Overall, these results confirmed that HCC progression was promoted by MYLK-AS1. Hence, MYLK-AS1 could be used as a potential prognostic and diagnostic biomarker on HCC patients.

The biological functions of lncRNAs are dependent on their unique subcellular localizations, as demonstrated by several studies [28]. The cytoplasmic lncRNAs are known to act as mRNA decoys to influence the signal transduction pathways and in the regulation of the mRNA translation or stability [24]. miRNAs in cancer play a key role in signal transduction by inhibiting or degrading mRNAs [29]. miR-424-5p is involved in the regulation of invasion, metastasis, apoptosis, and proliferation in a variety of cancer cells, as revealed by recent studies [30–33]. La ribonucleoprotein 4 (LARP4) acts as a molecular sponge of miR-424 in gastric cancer, in which a similar ceRNA related mechanism was reported [33]. Our results showed that MYLK-AS1 directly bound and inhibited miR-424-5p expression in the HCC cells, with miR-424-5p expression being significantly downregulated and negatively correlated with MYLK-AS1 expression, as confirmed by the RIP and luciferase reporter assays and the bioinformatics analysis. HCC progression was promoted by the low miR-424-5p expression due to the ceRNA nature of MYLK-AS1, thereby alleviating the downregulation of E2F7.

Tumor angiogenesis is a key mechanism that promotes tumor growth and invasion [34] also in HCC [35]. E2F7 directly stimulates and binds with the VEGFA promoter to influence tissue angiogenesis [36]. Besides, the involvement of E2F7 in the progression of HCC is also confirmed by numerous studies [37–39]. The present study revealed that E2F7 was a direct target of miR-424-5p and the loss of the E2F7 inhibition by miR-424-5p significantly enhanced VEGFR-2 pathway activity. At the same time, the VEGFR-2 signaling pathway was significantly inactivated in HCC cells with low MYLK-AS1 expression. The other 5 mRNAs (CBX2, CLSPN, CCNE1, CEP55, and CDC25A) besides E2F7, also containing a potential miR-424-5p binding site, were also significantly downregulated in HCC samples obtained from TCGA database. Nonetheless, the results of the preliminary experiments revealed that E2F7 and miR-424-5p had the strongest ability of binding each other. Hence, our hypothesis was that miR-424-5p regulates E2F7 expression in HCC. Our co-culture of HCC cells with HUVECs supported the evidence that MYLK-AS1 and E2F7 are involved in the promotion of vascular endothelial cell proliferation, further demonstrating its importance in angiogenesis. Comprehensively, our findings demonstrated that the MYLK-AS1/miR-424-5p axis activated the VEGFR-2 signaling pathway by specifically upregulating E2F7 expression.

Our results showed that VEGFR-2 was mainly expressed on the cell membrane and was found on both HCC cell lines and vascular endothelial cells. The E2F7 protein was mainly found in the cytoplasm and it is associated with tissue angiogenesis [36] and with the transcriptional regulation of the DNA damage-dependent cell-cycle genes [40]. Our findings revealed that E2F7 protein was transported outside the HCC cells, maybe by exosomes, acting on the tumor microenvironment to increase the angiogenesis of the HCC tissue, enhancing the metastasis and proliferation of the HCC cells (Fig. 8b). In addition, this study highlighted the role of MYLK-AS1 as a ceRNA in the binding of miR-424-5p and that the upregulation of E2F7 resulted in the increased activation of VEGFR-2 signaling pathway, promoting tumor angiogenesis, proliferation, and metastasis. Nonetheless, the exact drivers responsible for the change in the location of E2F7 and the specific pathway and mechanism of E2F7 to escape out from HCC and act on the surrounding tissues and vascular endothelial cells remain to be discovered. Despite our results elucidating the role of MYLK-AS1 in HCC development and angiogenesis, E2F7-VEGFR2 interaction should be further analyzed.

## Conclusion

In conclusion, this study demonstrated that lncRNA MYLK-AS1 upregulation was frequent in HCC. MYLK-AS1 promoted HCC tumor angiogenesis and cell proliferation in vitro and in vivo by directly targeting miR-424-5p to upregulate E2F7 and activate VEGFR-2 signaling, consequently promoting the HCC progression. Thus, MYLK-AS1 could be considered as a new prognostic biomarker and therapeutic target in HCC.

### Supplementary information

**Additional file 1: Table S1.** Primer and oligonucleotide sequences used in this study.

**Additional file 2: Figure S1.** MYLK-AS1 expression in HCC patients in different clinical subgroups. **A-C** Relative MYLK-AS1 expression in HCC with different tumor differentiation, vascular invasion, and with/without TACE treatment. Results were presented as the relative expression (compare to internal control, the 2-△△CT method) in tumor tissues and normal tissues. **D** ROC analysis of the different subgroups regarding the clinicopathological characteristics in patients with HCC. **E** MYLK-AS1 expression in 156 HCC tissues by qRTPCR. Relative MYLK-AS1 expression presented as the relative expression (compare to internal control, the 2-△△CT method) in the tumor tissues and matched normal tissues. HCC patients with TACE treatment were divided into high (*n* = 36) and low (*n* = 36) groups according to the median value (0.50).

## Data Availability

All data in our study are available upon request.

## Abbreviations

HCC: Hepatocellular carcinoma
lncRNAs: Long non-coding RNAs
qRT-PCR: Quantitative reverse transcription PCR
RIP: RNA binding protein immunoprecipitation
TCGA: The cancer genome atlas
ceRNA: Competing endogenous RNA
FISH: Fluorescence in situ hybridization
E2F7: E2F transcription factor 7
MYLK-AS1: MYLK antisense RNA 1
miRNAs: microRNAs
HUVECs: Human umbilical vein endothelial cells
LIHC: Liver hepatocellular carcinoma
FC: Fold change
FDR: False discovery rate
AJCC: American joint committee on cancer
HEK: Human embryonic kidney
IHC: Immunohistochemistry
H-score: Histoscore
shRNAs: Short-hairpin RNAs
NC: Negative control
PI: Propidium iodide
DIG: Digoxigenin
HRP: Horseradish peroxidase
CDC25A: Cell division cycle 25A
EZH2: Enhancer of zeste 2 polycomb repressive complex 2 subunit
CBX2: Chromobox 2
KIF23: Kinesin family member 23
SLC7A11: Solute Carrier family 7 member 11
