# Correction to: LncRNA MYLK-AS1 facilitates tumor progression and angiogenesis by targeting miR-424-5p/E2F7 axis and activating VEGFR-2 signaling pathway in hepatocellular carcinoma

**DOI:** 10.1186/s13046-020-01780-y

**Published:** 2020-12-09

**Authors:** Fei Teng, Ju-Xiang Zhang, Qi-Meng Chang, Xu-Bo Wu, Wei-Guo Tang, Jian-Fa Wang, Jin-Feng Feng, Zi-Ping Zhang, Zhi-Qiu Hu

**Affiliations:** 1 Department of Hepatobiliary and Pancreatic Surgery, Minhang Hospital, Fudan University, Shanghai, 201199 People’s Republic of China; 2 Institute of Fudan-Minhang Academic Health System, Minhang Hospital, Fudan University, Shanghai, 201199 People’s Republic of China; 3 Shanghai Med-X Engineering Center for Medical Equipment and Technology, School of Biomedical Engineering, Shanghai Jiao Tong University, Shanghai, 200030 People’s Republic of China

**Correction to: J Exp Clin Cancer Res 39, 235 (2020)**

**https://doi.org/10.1186/s13046-020-01739-z**

Following publication of the article [1], the authors identified errors in Figure 1g,1i,1j,1k, Figure 5e,f, and Figure 6d,e. In addition, figure captions for Figure 2, Figure 4, Figure 5, Figure 6, Figure 7, and Additional file 2: Figure S1 should be corrected. The corrected figures and captions can be found below. The corrections do not change the results or the conclusions of this article.

The original article has been updated.

**Fig. 2 MYLK-AS1 and E2F7 overexpression is positively correlated with HCC progression and poor prognosis. A-C** Relative expression of MYLK-AS1, miR-424-5p and E2F7 detected by qRT-PCR in 156 paired HCC cancer tissues and matched normal liver tissues. Results are presented as the relative expression (compare to internal control, the 2^-△△CT^ method) in tumor tissues and peritumoral tissues. **D** MYLK-AS1 expression in peritumoral tissues and HCC tissues by ISH. **E** Relative MYLK-AS1 expression by qRT-PCR in 156 HCC tissues. Relative MYLK-AS1 expression presented as the relative expression (compare to internal control, the 2^-△△CT^ method) in tumor tissues and the matched normal tissues. HCC patients were divided into high (n = 78) and low (n = 78) group according to the median value (0.50). **F-G** Relative MYLK-AS1 expression in HCC with different size and stage. Results were presented as the relative expression (compare to internal control, the 2^-△△CT^ method) in tumor tissues and normal tissues. **H-I** Kaplan-Meier plots of the OS and PFS of HCC patients with high (n = 78) and low (n = 78) MYLK-AS1 expression. Data are presented as mean ± SD. *P < 0.05, **P < 0.01, and ***P < 0.001. **J** ROC analysis of the performance of MYLK-AS1 expression of 1-year OS in all patients. **K** Kaplan-Meier plots of the PFS of HCC patients after postoperative adjuvant TACE therapy with high (n = 36) and low (n = 36) MYLK-AS1 expression. Data are presented as mean ± SD. *P < 0.05, **P < 0.01, and ***P < 0.001.

**Fig. 4 MYLK-AS1 up-regulates E2F7 expression by competitively binding miR-424-5p in HCC. A-B** Binding ability of MYLK-AS1, miR-424-5p, and E2F7 to anti-Ago2 in Hep-G2 and MHCC-97H (anti-igG was used as control) by RIP assay. **C** HEK-293FT cells co-transfected with wide type (WT) or mutated (Mut) E2F7 3’-UTR reporter vector and miR-424-5p mimic by luciferase reporter assay. **D** Relative E2F7 mRNA expression in Hep-G2 cells after MYLK-AS1 knockdown or in MHCC-97H cells after MYLK-AS1 overexpression. **E** E2F7 mRNA expression in MHCC-97H cells after reducing the expression of MYLK-AS1 and/or inhibition of miR-424-5p by qRT-PCR. **F** E2F7 mRNA expression in MHCC-97H cells following the ectopic expression of miR-424-5p and/or pLVX-E2F7 expression vector lacking the 3’-UTR by qRT-PCR. Results are expressed as mean ± SD. n.s, not significant, *P < 0.05, **P < 0.01, and ***P < 0.001

**Fig. 5 Reduced expression of MYLK-AS1 decreases the proliferation, migration and invasion of HCC cells. A** Relative MYLK-AS1 expression in Hep-G2 and MHCC-97H cells transfected with two independent shRNAs targeting MYLK-AS1 by qRT-PCR. **B-C** Hep-G2 and MHCC-97H cell proliferation after knockdown of MYLK-AS1 by CCK-8 assay. **D-H** Representative results of the colony formation (scale bar = 500 μm), cell cycle assay, apoptosis assays, and transwell assay (scale bar = 100 μm), in Hep-G2 and MHCC-97H cells after shMYLK-AS1–1 or shMYLK-AS1–2 transfection. Results are expressed as mean ± SD. *P < 0.05, **P < 0.01, and ***P < 0.001

**Fig. 6 Overexpression of MYLK-AS1 promotes the proliferation, migration and invasion of HCC cells. A** Relative MYLK-AS1 expression in Hep-G2 and MHCC-97H cells after MYLK-AS1 overexpression by qRT-PCR. **B** Proliferation of Hep-G2 and MHCC-97H cells after MYLK-AS1 overexpression by CCK-8 assay. **D-E** Representative results of the cell cycle and apoptosis assay of Hep-G2 and MHCC-97H cells after MYLK-AS1 overexpression. **F-G** Migration and invasion of Hep-G2 and MHCC-97H cells after MYLK-AS1 overexpression by transwell assay (scale bar = 100 μm). Results are expressed as mean ± SD. *P < 0.05, **P < 0.01, and ***P < 0.001

**Fig. 7 MYLK-AS1 regulates HCC cell proliferation and angiogenesis** ***in vivo and in vitro***. **A** Injection in the right armpit of MHCC-97H cells transfected with empty vector or MYLK-AS1 expression vector and shMYLK-AS1-NC or shMYLK-AS1-2 in the upper panel. Representative images of xenograft tumors are shown in the bottom panel. **B-C** Tumor weight and volume of the xenograft in MYLK-AS1 overexpression groups and control group or MYLK-AS1 knockdown group and control group. **D** Representative IHC staining results of CD34 in corresponding xenografts (scale bar = 50 μm). **E-F** Statistical analysis of the H-score of CD34 in the corresponding xenografts. Results are presented as mean ± SD from three independent experiments. **G** Cell proliferation of HUVECs cells cultured alone and co-cultured with MHCC-97H cells by CCK-8. **H** MYLK-AS1 knocked down or overexpressed or E2F7 overexpressed MHCC-97H cells co-cultured with HUVECs cells and consequent HUVEC proliferation by CCK-8. Results are expressed as mean ± SD. *P < 0.05, **P < 0.01, and ***P < 0.001

**Additional file 2: Fig. S1. MYLK-AS1 expression in HCC patients in different clinical subgroups. A-C** Relative MYLK-AS1 expression in HCC with different tumor differentiation, vascular invasion, and with/without TACE treatment. Results were presented as the relative expression (compare to internal control, the 2^-△△CT^ method) in tumor tissues and normal tissues. **D** ROC analysis of the different subgroups regarding the clinicopathological characteristics in patients with HCC. **E** MYLK-AS1 expression in 156 HCC tissues by qRT-PCR. Relative MYLK-AS1 expression presented as the relative expression (compare to internal control, the 2^-△△CT^ method) in the tumor tissues and matched normal tissues. HCC patients with TACE treatment were divided into high (n = 36) and low (n = 36) groups according to the median value (0.50).

**Fig. 1:**
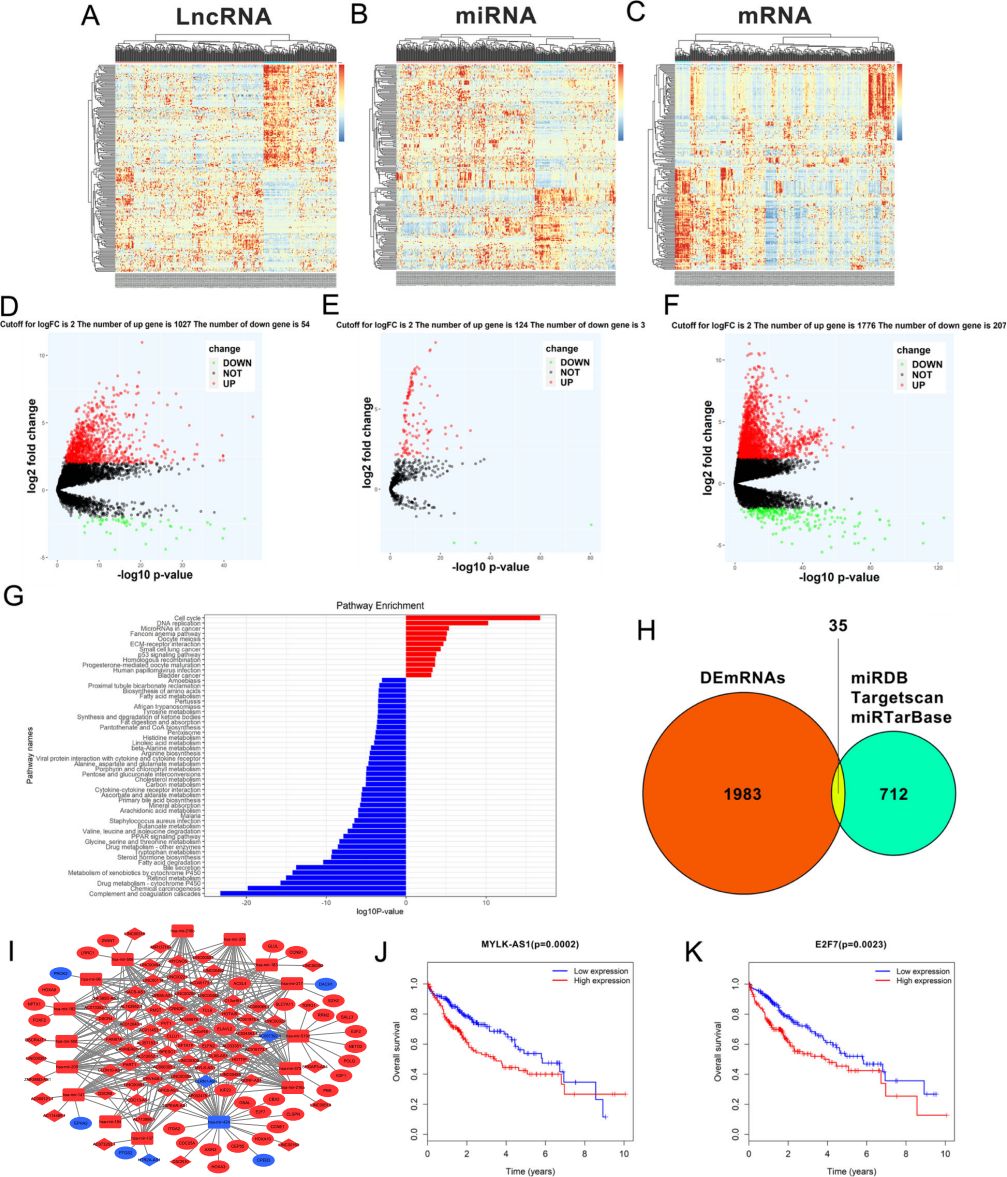
RNA-seq data analysis of HCC in TCGA database. **a**-**c** Clustered heat maps of the differentially expressed RNAs in HCC tissues and adjacent non-tumor liver tissues. Rows represent RNAs, whereas columns represent HCC tissues and adjacent non-tumor liver tissues. Differentially expressed lncRNAs, miRNAs, and mRNAs in HCC tissues compared to adjacent non-tumor liver tissues were filtered by standard of log2FC > 2 and FDR < 0.01. FC: folds change; FDR: false discovery rate. **d**-**f** Volcano plots visualizing and assessing the variation of (D) long non-coding RNAs, (E) microRNAs, and (F) mRNAs expression between HCC tissues and adjacent non-tumor liver tissues. The values of the X-axis the indicate 10log p value and the Y-axis indicate the log2 fold change of the group. **g** Enriched KEGG pathways for differentially expressed mRNAs (the bar plot shows the enrichment scores of the significantly enriched KEGG pathways). KEGG, Kyoto Encyclopedia of Genes and Genomes. **h** Identification of 712 changed targeted mRNAs among the 127 DEmiRNAs from the three public profile datasets (miRDB, Targetscan and miRTarBase). The cross areas revealed that the number of commonly changed mRNAs between DEmRNAs and target mRNAs was 35, which included E2F7. **i** The lncRNA-miRNA-mRNA ceRNA network. Blue squares, downregulated miRNAs; blue circles, downregulated mRNAs; blue diamonds, downregulated lncRNAs. Red squares, upregulated miRNAs; red circles, upregulated mRNAs; red diamonds, upregulated lncRNAs. **j**-**k** Kaplan-Meier survival curves for MYLK-AS1 and E2F7 associated with OS

**Fig. 5:**
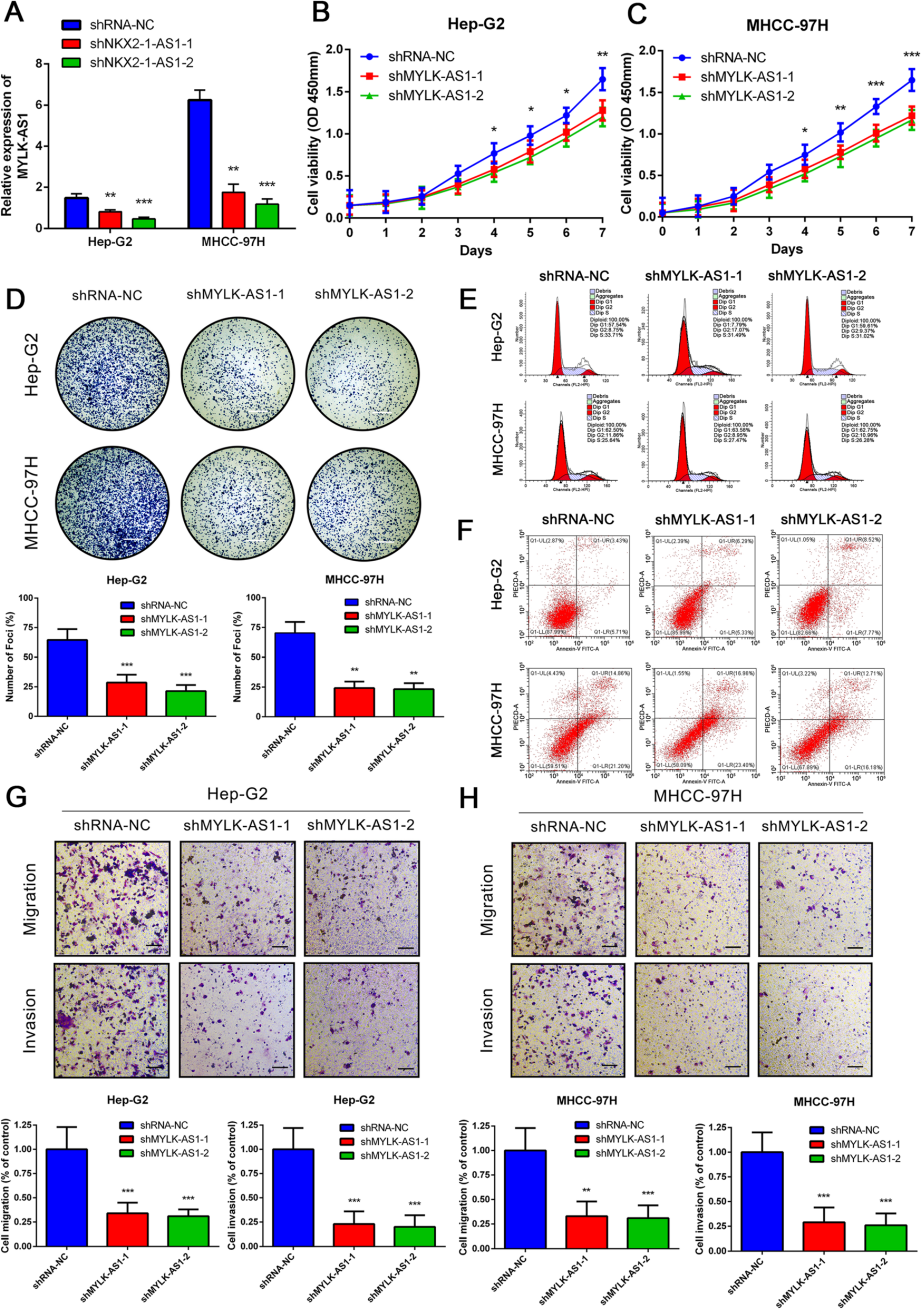
Reduced expression of MYLK-AS1 decreases the proliferation, migration and invasion of HCC cells. **a** Relative MYLK-AS1 expression in Hep-G2 and MHCC-97H cells transfected with two independent shRNAs targeting MYLK-AS1 by qRT-PCR. **b**-**c** Hep-G2 and MHCC-97H cell proliferation after knockdown of MYLK-AS1 by CCK-8 assay. **d**-**h** Representative results of the colony formation (scale bar = 500 μm), cell cycle assay, apoptosis assays, and transwell assay (scale bar = 100 μm), in Hep-G2 and MHCC-97H cells after shMYLK-AS1–1 or shMYLK-AS1–2 transfection. Results are expressed as mean ± SD. *P < 0.05, **P < 0.01, and ***P < 0.001

**Fig. 6:**
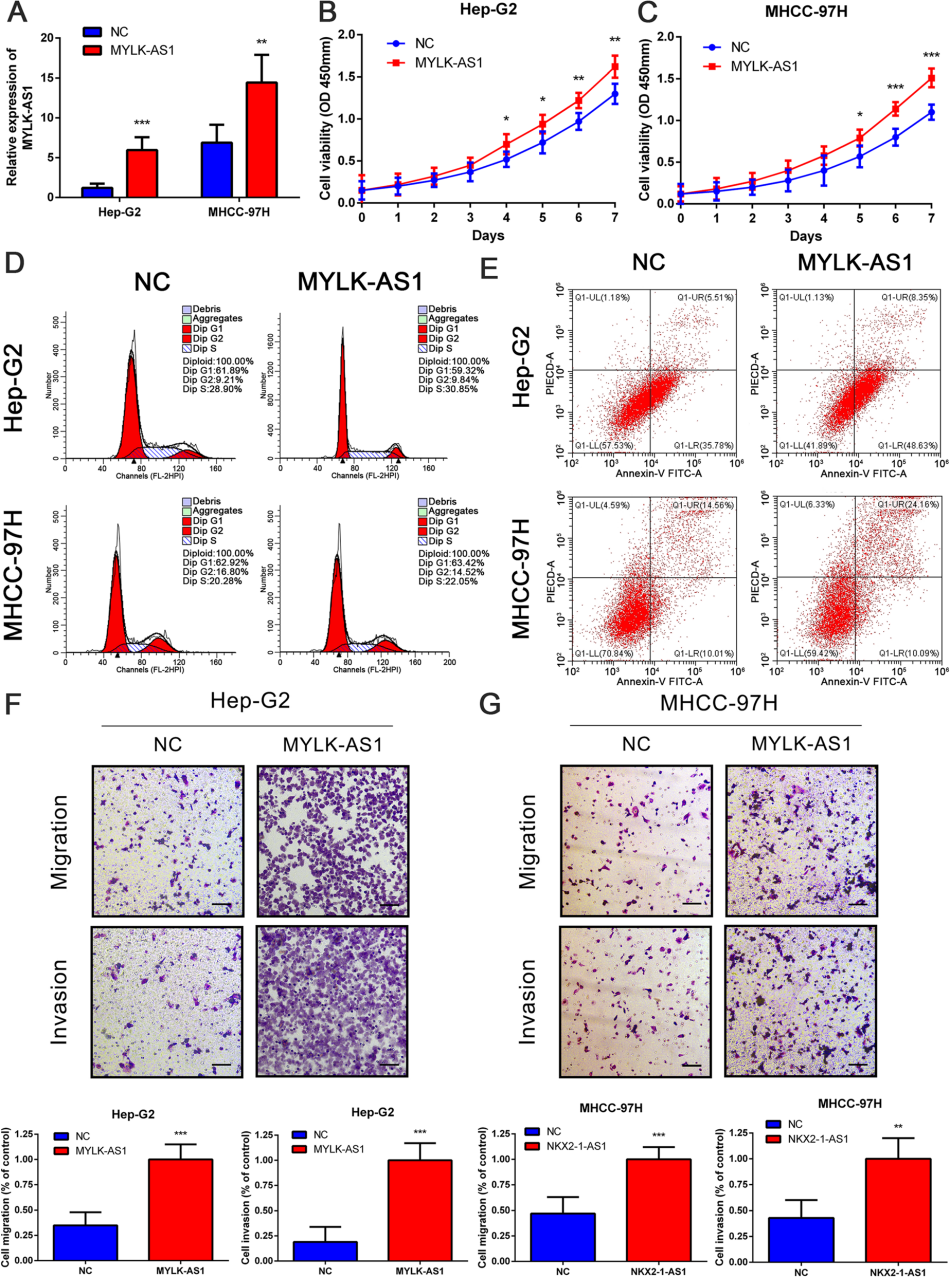
Overexpression of MYLK-AS1 promotes the proliferation, migration and invasion of HCC cells. **a** Relative MYLK-AS1 expression in Hep-G2 and MHCC-97H cells after MYLK-AS1 overexpression by qRT-PCR. **b** Proliferation of Hep-G2 and MHCC-97H cells after MYLK-AS1 overexpression by CCK-8 assay. **d**-**e** Representative results of the cell cycle and apoptosis assay of Hep-G2 and MHCC-97H cells after MYLK-AS1 overexpression. **f**-**g** Migration and invasion of Hep-G2 and MHCC-97H cells after MYLK-AS1 overexpression by transwell assay (scale bar = 100 μm). Results are expressed as mean ± SD. *P < 0.05, **P < 0.01, and ***P < 0.001
