# Correction: LncRNA MYLK-AS1 facilitates tumor progression and angiogenesis by targeting miR-424-5p/E2F7 axis and activating VEGF R-2 signaling pathway in hepatocellular carcinoma

**DOI:** 10.1186/s13046-023-02824-9

**Published:** 2023-09-30

**Authors:** Fei Teng, Ju-Xiang Zhang, Qi-Meng Chang, Xu-Bo Wu, Wei-Guo Tang, Jian-Fa Wang, Jin-Feng Feng, Zi-Ping Zhang, Zhi-Qiu Hu

**Affiliations:** 1 Department of Hepatobiliary and Pancreatic Surgery, Minhang Hospital, Fudan University, Shanghai, 201199 People’s Republic of China; 2 2Institute of Fudan-Minhang Academic Health System, Minhang Hospital, Fudan University, Shanghai, 201199 People’s Republic of China; 3 Med-X Engineering Center for Medical Equipment and Technology, School of Biomedical Engineering, Shanghai Jiao Tong University, Shanghai, 200030 People’s Republic of China

**Correction:** ***J Exp Clin Cancer Res*** **39, 235 (2020)**

10.1186/s13046-020-01739-z

Following publication of the original article [1], incorrect Additional File 1 has been uploaded. The correct file replacement is now processed and uploaded.

The correction does not affect the overall result or conclusion of the article. The original article has been corrected.

### Electronic supplementary material

Below is the link to the electronic supplementary material.

**Table S1**. Primers and oligonucleotides sequences used in this study.

## References

[1] Teng F, Zhang JX, Chang QM (2020). LncRNA MYLK-AS1 facilitates tumor progression and angiogenesis by targeting miR-424-5p/E2F7 axis and activating VEGFR-2 signaling pathway in hepatocellular carcinoma. J Exp Clin Cancer Res.

