# Supplementary material for: LncRNA MYLK-AS1 facilitates tumor progression and angiogenesis by targeting miR-424-5p/E2F7 axis and activating VEGFR-2 signaling pathway in hepatocellular carcinoma
Source: J Exp Clin Cancer Res. 2020 Nov 9;39:235. doi: 10.1186/s13046-020-01739-z (PMC7650167; doi:10.1186/s13046-020-01739-z)
Supplement: Supplementary file 1 — Additional file 1: Table S1. Primer and oligonucleotide sequences used in this study. [file 13046_2020_1739_MOESM1_ESM.pdf]

Table S1. Primers and oligonucleotides sequences used in this study

Name	Sequence 5'-3'
MYLK-AS1-forward	GAATCCAGTGTCGTCCGTGA
MYLK-AS1-reverse	AGGCAGATACCTGGCTTCCA
MYLK-AS1-shRNA-1-forward	CCGGAGAATCCAGTGTCGTCCGTGCTCGAGCACGGACGACACTGGATTCTTTTTG
MYLK-AS1-shRNA-1-reverse	AATTCAAAAAAGAATCCAGTGTCGTCCGTGCTCGAGCACGGACGACACTGGATTCTCCGG
MYLK-AS1-shRNA-2-forward	CCGGGAGAGTGAGGTGCGGGATTGCTCGAGCAATCCCGCACCTCACTCTCTTTTTG
MYLK-AS1-shRNA-2-reverse	AATTCAAAAAGAGAGTGAGGTGCGGGATTGCTCGAGCAATCCCGCACCTCACTCTCCCGG
MYLK-AS1-FISH probe	CAGTGAGATTGGAAGCCAGGT
E2F7-forward	CTGCTGCGCTAGACTTGGATA
E2F7-reverse	AACCCTGGTCAGTGTAGGGC
miR-424-5p-forward	CGAGGGGATACAGCAGCAAT
miR-424-5p -reverse	TTCCCCACGAGGGGGTATAG
U6 snRNA-forward	AAAGCAAATCATCGGACGACC
U6 snRNA-reverse	GTACAACACATTGTTTCCTCGGA
GAPDH-forward	GAAAGCCTGCCGGTGACTAA
GAPDH-reverse	TTCCCGTTCTCAGCCTTGAC
