# Supplementary figures and images for: LncRNA MYLK-AS1 facilitates tumor progression and angiogenesis by targeting miR-424-5p/E2F7 axis and activating VEGFR-2 signaling pathway in hepatocellular carcinoma
Source: J Exp Clin Cancer Res. 2020 Nov 9;39:235. doi: 10.1186/s13046-020-01739-z (PMC7650167; doi:10.1186/s13046-020-01739-z)

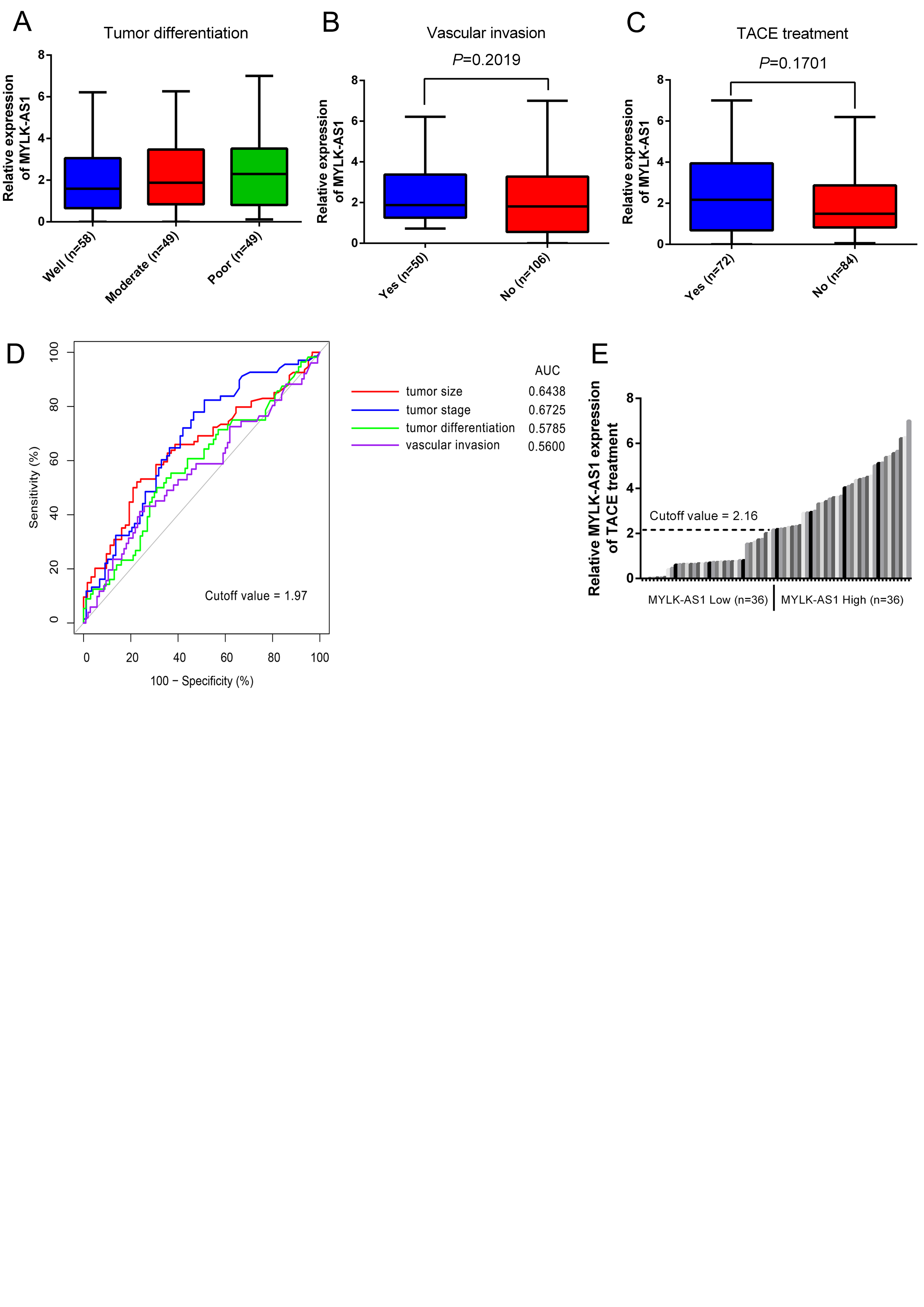

Supplement: Supplementary file 2 — Additional file 2: Figure S1. MYLK-AS1 expression in HCC patients in different clinical subgroups. A-C Relative MYLK-AS1 expression in HCC with different tumor differentiation, vascular invasion, and with/without TACE treatment. Results were presented as the relative expression (compare to internal control, the 2-△△CT method) in tumor tissues and normal tissues. D ROC analysis of the different subgroups regarding the clinicopathological characteristics in patients with HCC. E MYLK-AS1 expression in 156 HCC tissues by qRTPCR. Relative MYLK-AS1 expression presented as the relative expression (compare to internal control, the 2-△△CT method) in the tumor tissues and matched normal tissues. HCC patients with TACE treatment were divided into high (n = 36) and low (n = 36) groups according to the median value (0.50). [file 13046_2020_1739_MOESM2_ESM.tif]
